# 1,8-diiodooctane acts as a photo-acid in organic solar cells

**DOI:** 10.1038/s41598-019-40948-1

**Published:** 2019-03-13

**Authors:** Nutifafa Y. Doumon, Gongbao Wang, Xinkai Qiu, Adriaan J. Minnaard, Ryan C. Chiechi, L. Jan Anton Koster

**Affiliations:** 1 Photophysics and OptoElectronics, Zernike Institute for Advanced Materials, University of Groningen, Nijenborgh 4, NL-9747 AG Groningen, The Netherlands; 2 Stratingh Institute for Chemistry, University of Groningen, Nijenborgh 4, NL-9747 AG Groningen, The Netherlands

## Abstract

The last decade saw myriad new donor polymers, among which benzodithiophene-co-thienothiophene polymers are attractive due to their relatively high power conversion efficiency in bulk heterojunction solar cells. We examine the effect of UV-light on the stability of these polymers. The relationship between the polymer chemical structure and the UV-stability of the cells is explored on the one hand, and on the other hand, the effect of additives on their UV-stability: 1,8-diiodooctane against 1-chloronaphthalene in the cells and 1,8-octanedithiol in solution. For example, PBDTTT-E with 18% efficiency loss is more stable than PBDTTT-ET with 36% loss throughout the exposure. While 1,8-diiodooctane acts as photo-acid and leads to accelerated degradation of the solar cells, 1-chloronaphthalene does not. Acidity is known to be detrimental to the efficiency and stability of organic solar cells. The degradation is initiated upon UV-irradiation by the cleavage of the side chains, resulting in more electron traps and by the formation of iodine, dissolved HI and carbon-centered radicals from 1,8-diiodooctane as revealed by ^1^H NMR spectrum. The 1,8-octanedithiol spectra do not show such species. Finally, the mechanisms behind the effect of 1,8-diiodooctane are explained, paving the way for the design of new, efficient as well as stable materials and additives.

## Introduction

Over the past few decades, polymer solar cells have received attention in the scientific community due to their potential, though debatable, of becoming cheaper alternatives to the existing solar cell technologies. This notion is mainly driven by the fact that these devices are easily solution-processed^1^, giving the possibility for eventual large-scale fabrication (of both small and large area devices) and flexible solar cell technologies. Efforts to boost this technology were mostly limited to the improvement of device efficiency, while relatively little has been done on the device stability and lifetime^2–7^. Device degradation is a complex mechanism, as many factors play key but different roles in the process. Poly(3-hexylthiophene), P3HT, one of the workhorse polymers has extensively been studied and was found to be relatively stable under thermal stress, continuous illumination and in ambient conditions^8,9^. However, P3HT solar cells have low efficiency^10^. Thus, new materials that would yield highly efficient, as well as stable devices, are needed. In this regard, myriad polymers including the benzodithiophene (BDT) novel polymers were structurally engineered^11–20^. The series of benzodithiophene-co-thienothiophene (BDT-TT) polymers were subsequently synthesized of which among the alkoxy-BDT (1D) polymers, Poly[[4,8-bis[(2-ethylhexyl)oxy]benzo[1,2-b:4,5-b′]dithiophene-2,6-diyl][3-fluoro-2-[(2-ethylhexyl)carbonyl]thieno[3,4-b]thiophenediyl]], PTB7, quickly became the workhorse material^21^. Subsequently, the alkylthienyl (2D) polymers of the family were engineered giving rise to an improvement in the efficiency of their devices. The provision of these novel materials, combined with device architecture and processing techniques yielded power conversion efficiencies (PCEs) above 10% for single junction polymer-fullerene cells^16,22,23^. A typical example is the combined use of additives (e.g. diiodooctane, DIO or octanedithiol, ODT) and the change in interfacial layers and electrodes in the use of inverted structure^24–29^. These strategies were combined recently to produce a power conversion efficiency (PCE) of 17.3%^30^. However, stability studies^2,5,6,23,31–33^ of these materials and their devices are limited, and more importantly, the mechanism behind the degradation is still only partially understood and needs more attention to be generalized to all-polymer solar cells. It is shown that additives, such as DIO, have conflicting effects on device performance, i.e. even though DIO aids in the improvement of device efficiency, it is an agent of accelerated degradation in the devices^34–36^. The reason behind this conflicting effect is little understood, tricky to elucidate, and thus has been sporadically explained in the literature vis-a-vis different materials^7,37,38^. While some think that DIO becomes an inherent part of the deposited film that it cannot be fully removed from the film^37,39,40^; others have it that it is possible to remove DIO by vacuum and/or thermal annealing and washing with methanol^7,41,42^.

**Figure 1:**
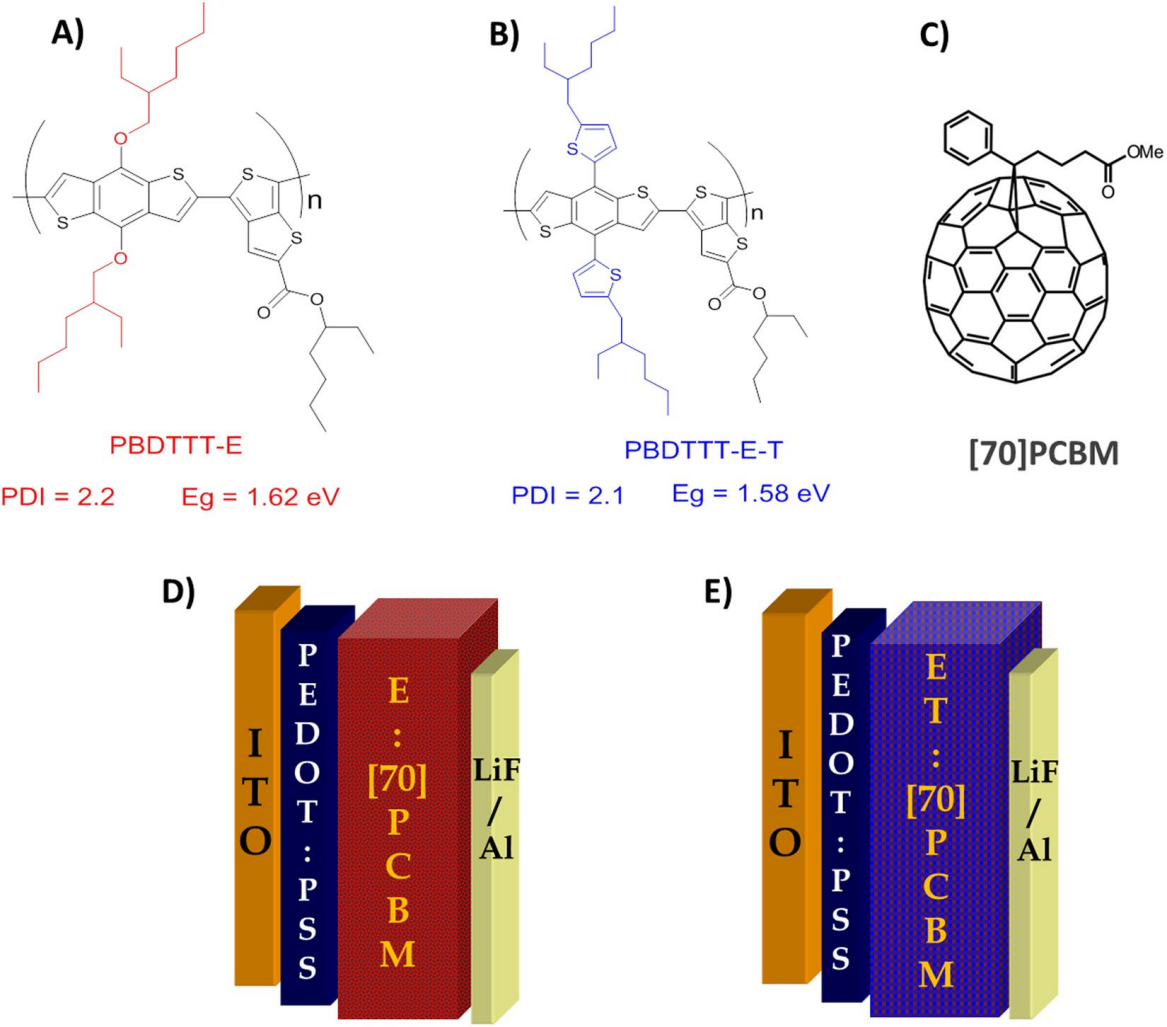
Chemical structure of materials used in the active blend layer: E polymer (**A**), ET polymer (**B**) with fullerene derivative [70]PCBM (**C**) and corresponding device structure of E blend (**D**) and ET blend (**E**).

This paper aims to show the relation between the degradation of polymer:fullerene solar cells and the chemical structure of the polymers, to identify why DIO is not good for device stability, and to explain the mechanism behind these effects for a pair of BDT-TT polymers that can be extrapolated. These are Poly[(4,8-bis-(2-ethylhexyloxy)-benzo(1,2-b:4,5-b′)dithiophene)-2,6-diyl-alt-((2-ethylhexyl)-thieno(3,4-b)thiophene-4-carboxylate))-2,6-diyl)], PBDTTT-E (hereafter E), a 1D polymer and its 2D polymer counterpart Poly[(4,8-bis(5-(2-ethylhexyl)thiophen-2-yl)-benzo[1,2-b;4,5-b]dithiophene)-2,6-diyl-alt-(4-(2-ethylhexyl)-thieno[3,4-b]thiophene-4-carboxylate))-2,6-diyl)], PBDTTT-ET (hereafter ET) with similar molecular weight and dispersity as shown in Fig. 1. The only difference between these two materials is in the side chain pendant group, with the former having alkoxy side chains (ether groups) on the BDT unit while the latter has alkylthienyl side chains (thiophene groups). In this work, we study the effect of their chemical structure on their photostability within the bulk heterojunction active layer of the solar cells processed with or without DIO (or CN), in an inert atmosphere. We take a particular look at the possible effect of the UV part of the solar spectrum on the stability of the devices, since the effects of other degradation agents have either been widely studied, as in the case of heat (thermal degradation) or can be carefully and easily avoided by encapsulation, in the case of oxygen (O_2_) and moisture (H_2_O). Our investigation focuses on the effect of DIO (or CN) and the difference in polymer chemical structure on PCE and the photostability of conventional solar cells under continuous illumination. The results show that ET cells processed with or without DIO degrade faster and thus, are less stable compared to E cells. This clearly shows that the alkylthienyl side chain polymer is more prone to degradation than the alkoxy ones under ultraviolet (UV) light. Charge transport and FTIR measurements were respectively studied on both the pristine polymers and blends with Phenyl-C71-butyric acid methyl ester, [70]PCBM, films to have a deep understanding of the observed degradation in the solar cells and its pathway. Finally, we moved a step further to explain why DIO is detrimental to the device stability and the mechanisms behind these observed trends for both polymers using combined results from current-voltage (*J-V*) characteristics, NMR, and FTIR measurements. Our goal is to understand the underlying chemistry of the DIO-induced degradation mechanism, as these additives that solve some of the laboratory-scale efficiency issues exacerbate the oxidative/photo-induced damage. We found that DIO acts as a photo-acid in the active layer of the solar cell, generating HI under illumination as compared to ODT. Acidity is known to be detrimental to the efficiency and stability of organic solar cells, and HI is a very strong acid (7 pKa units more acidic than HCl). And even the acidity of PEDOT:PSS^43–45^ can have deleterious effects on the active layer. In this case, the formation of HI inside the active layer upon UV-exposure is detrimental to the photo-stability of the devices.

## Results and Discussions

### Performance of Devices: Initial Power Conversion Efficiency

**Figure 2:**
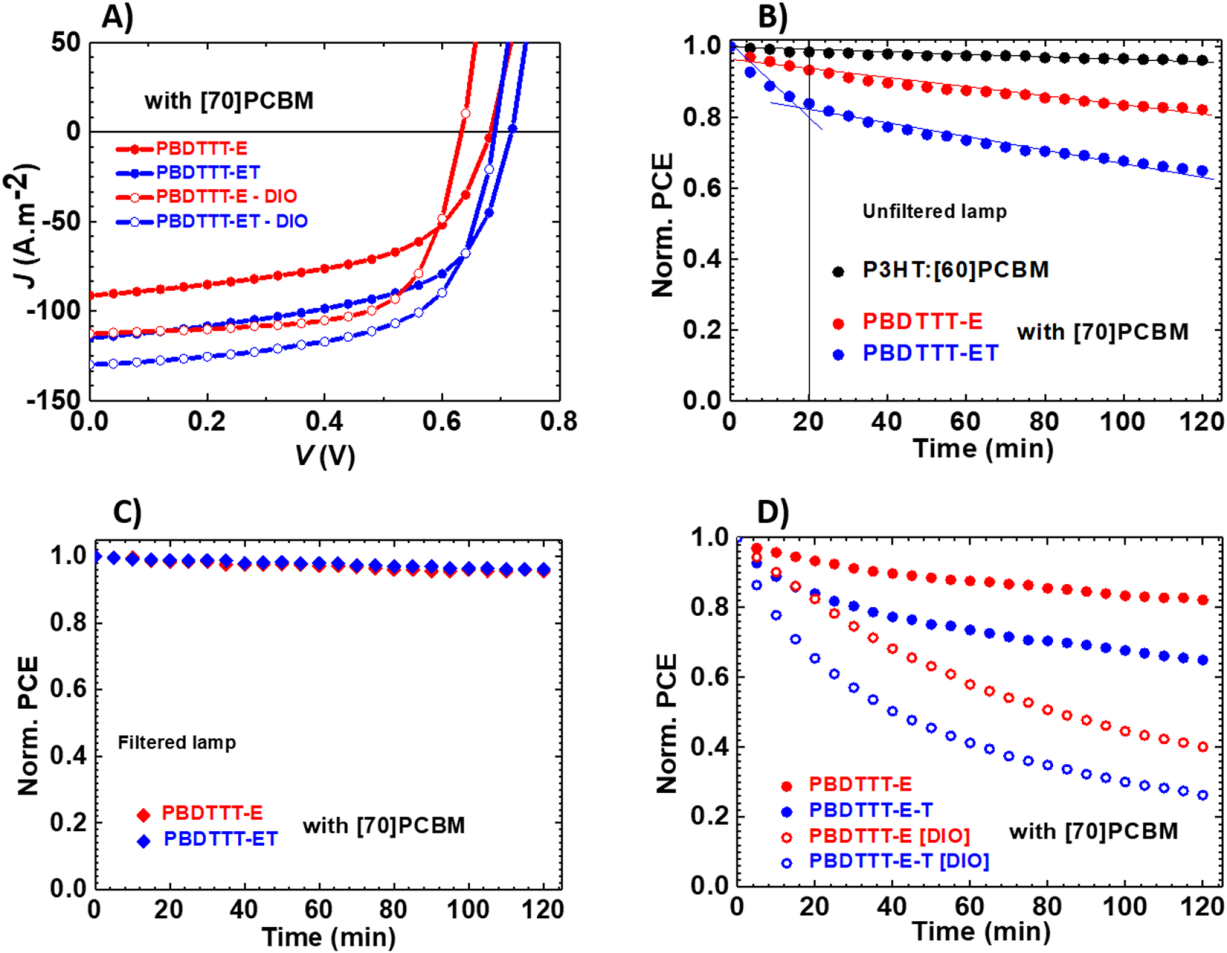
Performance of the cells with (open symbols) and without (full symbols) DIO under simulated 1 sun illumination using (un)filtered lamp: *J-V* characteristic curves of all cells under study (**A**); Evolution of PCE of P3HT:[60]PCBM, E:[70]PCBM and ET:[70]PCBM cells normalized to their initial values under unfiltered lamp (**B**,**D**) and filtered lamp (**C**).

Figure 2 reveals the general performance of the solar cells under study, notably the PCE in A, and photo-degradation in B, C and D. The *J-V* characteristics in Fig. 2A, together with their parameters as displayed in Table 1, show a better performance for ET over E with 24%, 6%, and 32% increase in short circuit current (*J*_*sc*_), open circuit voltage (*V*_*oc*_), and PCE respectively when one transits from the E cell to the ET cell. This improvement can certainly be explained by the combined effects of many factors. ET has a slightly lower band gap (Fig. S1C), resulting in a slightly broader absorption band with an onset slightly red-shifted compared to E as shown in Fig. S1F and translated into an increase in *J*_*sc*_. Compared to E, ET has an improved charge carrier mobility with *µ*_*h*_ in the order of 19 × 10^−4^ cm^2^V^−1^s^−1^ as against 8.5 × 10^−4^ cm^2^V^−1^s^−1^ for E as obtained by the space charge current limited measurements (see Table S1 in SI). ET blend films have an ideality factor (*n*) of 1.43 compared to that of E blend films which is 1.67 as shown in Table 1, suggesting fewer traps in ET blend films over E as reflected by a slightly more homogeneous film morphology for ET in Fig. S2B. The addition of DIO during device processing notably increases *J*_*sc*_ and fill factor (FF), and thus, generally improves the efficiencies of the device as shown in Table 1. In general, there was 28%, 23%, 48% increase in *J*_*sc*_, FF, and PCE respectively for the E solar cell while there was 5%, 12%, 14% increase in *J*_*sc*_, FF and PCE for the ET one. This improvement in efficiency upon addition of DIO, as observed in a previous report^20^, is attributed to a number of factors including a better miscibility of the polymer:fullerene phases in the blend layer as shown in Figs S2C,D and S3C,D with a slight improvement of carrier mobilities as shown in Table S1. This improvement in efficiency is also reminiscent of the drop in *n* from 1.67 to 1.43 for E cells and from 1.43 to 1.34 for ET cells in Table 1, suggesting reduction in trap-assisted recombination in the devices processed with DIO.

**Table 1:** Device parameters of cells: P3HT blended with [60]PCBM in CB and the rest blended with [70]PCBM in oDCB.

Polymer/Solvents	L (nm)	*J*_*sc*_ (A.m^−2^)	*V*_*oc*_ (V)	FF (%)	PCE (%)	PCE_avg_ (%)	*n*_lvo_	*n*_avg_
P3HT/CB	—	94.6	0.568	64.1	3.4	3.2 ± 0.3	—	—
PBDTTT-E/oDCB	100	102.3	0.686	55.4	3.9	3.6 ± 0.2	1.67	1.73 ± 0.08
PBDTTT-E/oDCB:DIO	100	131.0	0.646	68.0	5.8	5.0 ± 0.5	1.43	1.47 ± 0.04
PBDTTT-ET/oDCB	100	127.3	0.727	55.3	5.1	4.3 ± 0.4	1.43	1.49 ± 0.06
PBDTTT-ET/oDCB:DIO	100	133.9	0.702	61.9	5.8	5.7 ± 0.1	1.34	1.40 ± 0.06

The average values for the cells are obtained for at least twenty devices processed from oDCB and for ten devices processed from oDCB:DIO. The thickness of devices is around 100 nm. L (Thickness), avg (average), *n* (ideality factor), and _lvo_ (lowest value obtained). The errors are standard deviations.

### Performance of Devices: Stability and Lifetime

#### UV-Degradation of Conventional E and ET Polymer Solar Cells

Solar cells of E and ET are fabricated under the same conditions. The devices, kept at room temperature by active cooling, are characterised in an inert atmosphere in a glovebox, with both O_2_ and H_2_O levels kept below 0.1 ppm. The cells are continuously exposed, under open circuit condition, to both UV-filtered and unfiltered solar simulator lamp (with their spectra shown in Fig. S4) for two hours while the *J-V* characteristics are measured at a constant interval of time throughout the experiment. Figure 2C shows the photostability behaviour of the two polymer cells with filtered UV-light, exhibiting an outstanding level of stability comparable to that of P3HT full light exposed cell shown in Fig. 2B. In contrast, under unfiltered lamp illumination, both cells showed different trends in their UV-degradation behaviour. For the E polymer, there is a gradual decline in the initial PCE which is relatively slow throughout the experiment. However, for the ET polymer, the PCE decay is relatively fast in the first 20 minutes subsequently followed by a much slower and stabilised decline in PCE as depicted in Fig. 2B. The observed photoinduced degradation started from the side chains in the first few minutes due to the cleavage of the side chains^32^. Upon cleavage, the residual side chains formed decomposition products in the active layers of both cells with the thiophene groups less stable than the ether groups. This effect could be possibly due to the fact that the alkylthienyl BDT polymers are prone to absorb more in the UV region of the spectrum than alkoxy BDT polymers (Fig. S1C,D, and E), especially, in the wavelength range of 250–310 nm as shown by the absorption spectra of the BDT-monomers in solution in Fig. S1B. Over the exposure time, E exhibited a decay of 18% from its initial PCE while ET recorded 36% PCE degradation from its initial value. The device parameters shown in Fig. S5B revealed that ET suffered the most loss in *J*_*sc*_ and FF while E suffered the most loss in *J*_*sc*_. The photostability lifetime (T_80_) of the devices were determined to be <30 minutes for ET and a minimum of two hours for E. This confirms that E is more stable than ET. However, under the same conditions as shown in Fig. 2B, considering the 3% decay recorded by P3HT:[60]PCBM (1:1 in CB) solar cell from its initial PCE of 3.4%, both polymer solar cells can be considered unstable.

To further comprehend the mechanisms behind the degradation of the polymer solar cells, variations in nano-morphological structures, variations in absorption spectra of polymer blends, and charge transport within the active layers before and after exposure are studied through AFM, UV-Vis and FTIR spectroscopy and *J-V* characteristics measurements respectively. Morphological change such as phase separation or change in absorbance upon illumination such as photobleaching could be the factors contributing to the observed degradation. Figure S1B,D, and E show the absorption spectra of the BDT-monomers and those of the E polymer, the fullerene derivative, and their blend respectively. Both polymer and [70]PCBM absorb in the UV range of 250–425 nm. Thus, one would assume that any change in the spectrum of the blend films upon UV-exposure could be due mostly to the difference in the polymer structures. As shown in Fig. S1G, H, both blend films did not register any change in spectra before and after exposure, eliminating photobleaching as a plausible cause for the degradation, within the time limit of the experiment. The same conclusion is arrived at for morphological changes as seen in Figs S2, S3. This implies that though morphological changes cannot be entirely discarded, phase separation upon light exposure could not be the reason behind the differences in the observed photodegradation of E and ET polymer solar cells.

**Figure 3:**
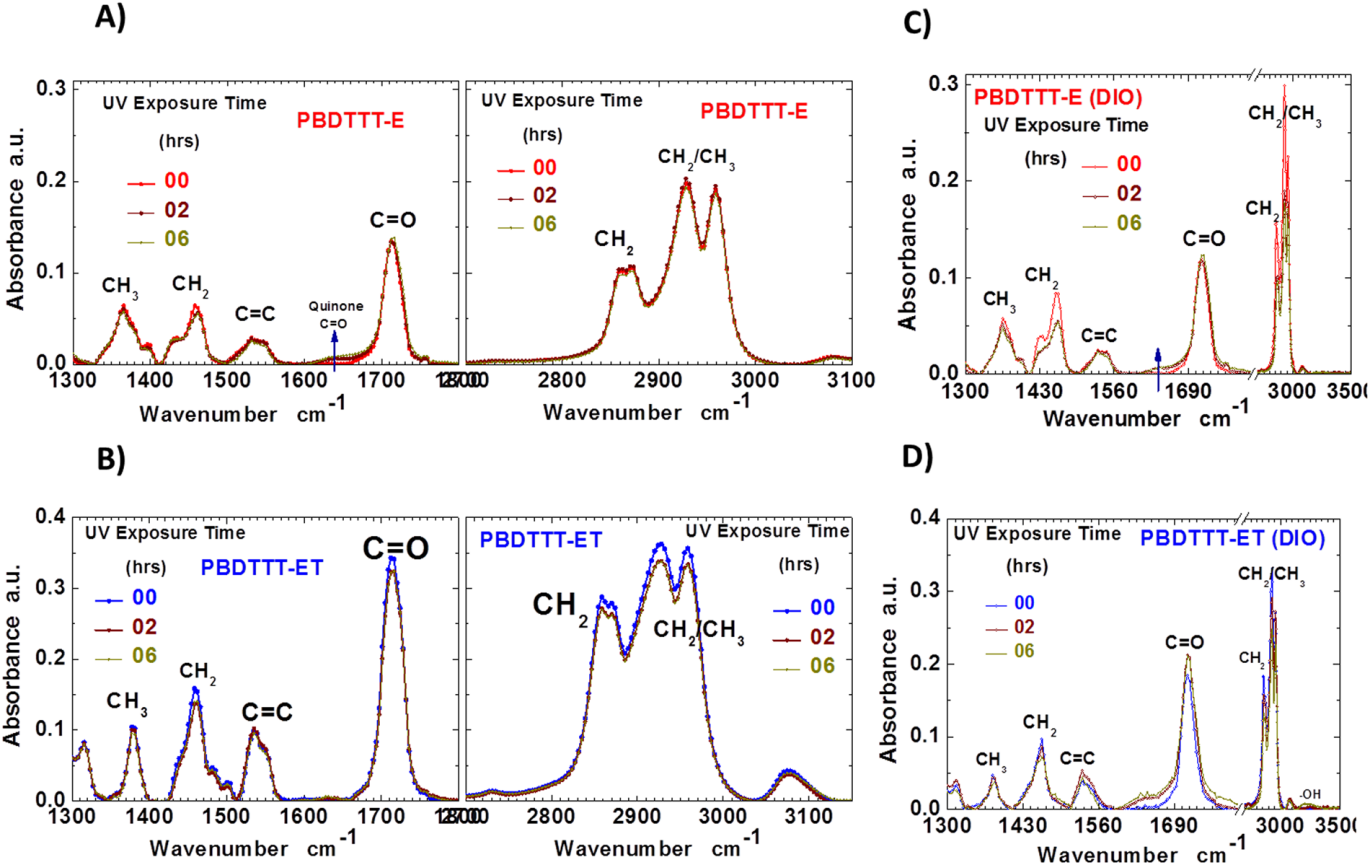
FTIR absorption spectra of unexposed and exposed (2 hours, 4 hours and 6 hours) films of pristine polymer films with (open symbols) and without (full symbols) DIO: E polymer without DIO (**A**) and with DIO (**C**); ET polymer without DIO (**B**) and with DIO (**D**).

Figures 3 and S5–S7 show the FTIR absorption profiles for drop cast films from a solution of [70]PCBM, both polymers, and both blends of comparable thickness before and after UV-exposure. The characteristic peaks of the absorption bands of the films are identified and assigned in Table S2. The peaks of the aliphatic side chains of the BDT-unit and the carbonyl group in the ester moiety for both pristine polymer films are observed between 2750–3000 cm^−1^ and 1714 cm^−1^ respectively while peaks characteristic of the thiophene rings are centred around 943 cm^−1^ and 1540 cm^−1^. From the spectra of the polymer films in Fig. 3, it is clear that the aliphatic side chains CH_2_, and CH_2_/CH_3_ around 2750–3000 cm^−1^ suffer the most reduction in intensity with the peaks of the ET side chains recording the higher reduction in intensity in Fig. 3B. Also, while all other peaks remained almost constant for the E polymer in Fig. 3A, there were noticeable reductions in some peaks of the ET polymer, as shown in Fig. 3B, notably at 1714 cm^−1^ characteristic of the carbonyl group and at the CH_2_ bending 1460 cm^−1^. This observation affirms our explanation that the degradation process is retarded in the E polymer cell compared to the ET polymer cell. This stabilised behavior of the E polymer could be due to the observed formation of benzoquinone^32^ as shown in Fig. 3A with a broad shoulder peak centered around 1640 cm^−1^ next to the ester carbonyl peak, as confirmed by recently published reports^38,46^. This possibly could be an explanation for the reduction of almost all other peaks in the ET system compared to the E system as also suggested by the PCE decay in Fig. 2B. This finding reiterates that the degradation is very dependent on the polymer chemical structure. The decomposition compounds from ET upon cleavage of the side chains are more volatile and destabilise their blend, and ultimately, their solar cell performance more than those of the E polymer. One would expect that if the exposure time were to be extended, the aliphatic chains peaks of ET would be the first to disappear totally. A look at [70]PCBM spectrum in Fig. S6C also revealed that PCBM went under degradation. The decrease in intensity of the absorbance in the same aliphatic and CH_2_ bending regions upon illumination was observed with an increase at the C=O stretching region while an initial increase followed by a significant decrease is recorded in the C=C stretching region. Together, in blend films, as shown in Fig. S6D,E the spectra demonstrated that E:[70]PCBM is more stable than ET:[70]PCBM. The spectra of the blends showed more peaks, characteristics of both polymer and fullerene, than those of the individual polymer films. There was, in general, a continuous decrease in signals of the aliphatic side chains and a sharp decrease in the peaks of the CH_2_ bending in the presence of [70]PCBM upon illumination, which was more pronounced for ET blend. In effect, two peaks can now be seen in the CH_2_ bending region namely at 1456 cm^−1^ characteristic of the polymers and 1430 cm^−1^ characteristic of the fullerene. Two peaks are also observed in the C=O region, one at 1714 cm^−1^ characteristic of the polymers and one at 1737 cm^−1^ characteristics of the fullerene. Upon illumination, while these peaks ostensibly remain constant for E:[70]PCBM films they revealed a gradual increase for the ET:[70]PCBM films. This is an indication of the presence of probable additional ester or carboxylic acid groups, formed from decomposition products of the ET blend films. There was an appearance of a new peak at 1573 cm^−1^ in both blend films at the initial stage, just next to the C=C stretch as can be seen in the E blend film. While this new peak completely disappeared upon illumination, there was a significant initial increase in the C=C stretch and appeared to be stabilized, a characteristic behaviour of the polymers as shown in their spectra between 1500–1570 cm^−1^ in Fig. 3A,B. This drastic change in the thienothiophene signal is more pronounced for the ET blend than the E blend (quite stabilised through benzoquinone formation). This could mean that the thiophene backbone rings/units were experiencing changes, possibly introduced by the decomposition products. It is evident that the ET:[70]PCBM films are less stable than the E:[70]PCBM ones.

**Figure 4:**
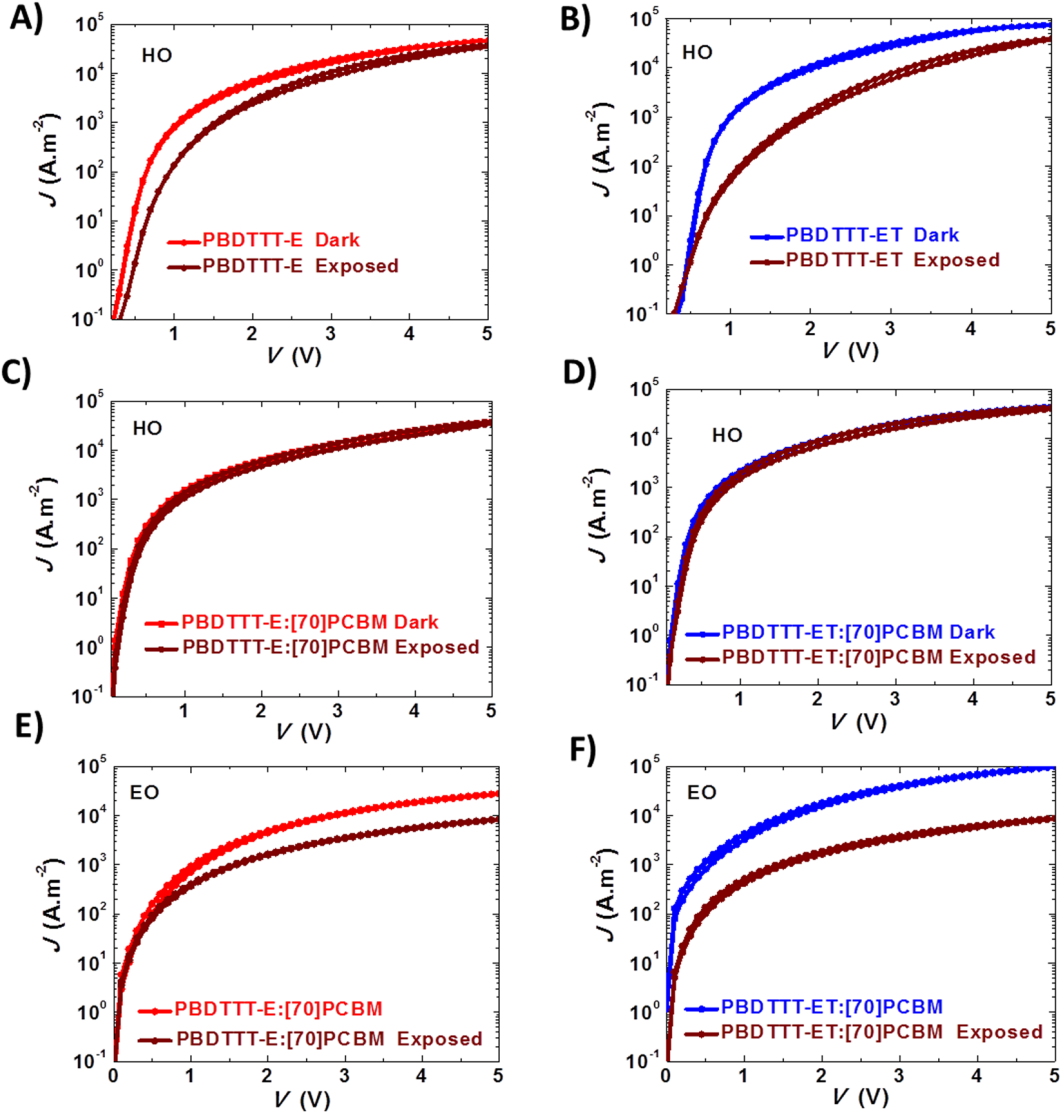
Current-voltage characteristics of unexposed (E, red and ET, blue) and exposed (1 hour - brown) of devices without DIO: Pristine polymer hole-only devices E and ET (**A**,**B**); blend hole-only devices E:[70]PCBM and ET:[70]PCBM (**C**,**D**); and blend electron-only devices E:[70]PCBM and ET:[70]PCBM (**E**,**F**).

With the confirmed role of the chemical structure of the polymer in the degradation process of the blends, one would expect to see the effect reflected in their charge transport. It is to be expected that the hole current reduces upon illumination for these systems. To prove this point, single carrier devices as described in the experimental section are fabricated. Figure 4 relays the *J-V* characteristics of as-cast (reference) and illuminated single carrier devices. Figure 4A,B show a decrease in the hole currents of the pristine polymers. This suggests that the polymers degrade upon UV exposure, with ET experiencing the larger loss in the hole current. The hole currents of the exposed and unexposed blends in Fig. 4C,D remained equal and limited by the hole current of the degraded pristine polymer. Finally, in Fig. 4E,F it is rather the electron currents in the blends that suffered a reduction in magnitude, totally consistent with our previous observation^32^. However, the reduction is more pronounced in ET blend (one order of magnitude in reduction) compared to E blend, suggesting a destabilisation of electron transport in the blend. These results strongly confirm the observation that the cleaved side chains and their subsequent breaking into smaller products, act as electron traps, with a more pronounced trap effect for ET than E. This is also evidenced by the reduction in electron mobilities of the blends, with almost two orders of magnitude reduction for ET from (1.2 × 10^−3^ cm^2^V^−1^s^−1^ to 5.0 × 10^−5^ cm^2^V^−1^s^−1^), compared with only close to 3 folds change for E (from 7.0 × 10^−4^ cm^2^V^−1^s^−1^ to 2.5 × 10^−4^ cm^2^V^−1^s^−1^) as shown in Table S1.

#### Effect of Diiodooctane on Stability and Lifetime of Conventional E and ET Polymer Solar Cells

DIO has a conflicting effect on the performance of the devices. First, a positive effect on morphology, that is a homogeneous miscibility of the polymer:fullerene blend irrespective of the polymer as shown in Fig. S2C, D, resulting in smaller *n* as compared to devices without DIO, and ultimately on PCE as shown in Fig. 2A (open symbols) and Table 1. Next, a negative effect on device stability, that is an accelerated UV-degradation upon illumination as seen in Fig. 2D. To have a better grasp of this negative effect, we conducted the same experiments on devices with DIO as in the case of devices without DIO. It emerged that DIO has a pronounced destabilization effect, coupled with the already discussed polymer chemical structure effect on the device stability. For example, in Fig. 2D it can be seen that the E polymer cell with DIO had a 60% decay in PCE (as opposed to 18%) while that of ET recorded 74% (as opposed to 36%), meaning while E remained more stable than ET, in this case, both had experienced pronounced degradation compared to the devices without DIO. Consequently, the E polymer cell with DIO had a T_80_ of 20 minutes while that of ET had a T_80_ of <10 minutes.

**Figure 5:**
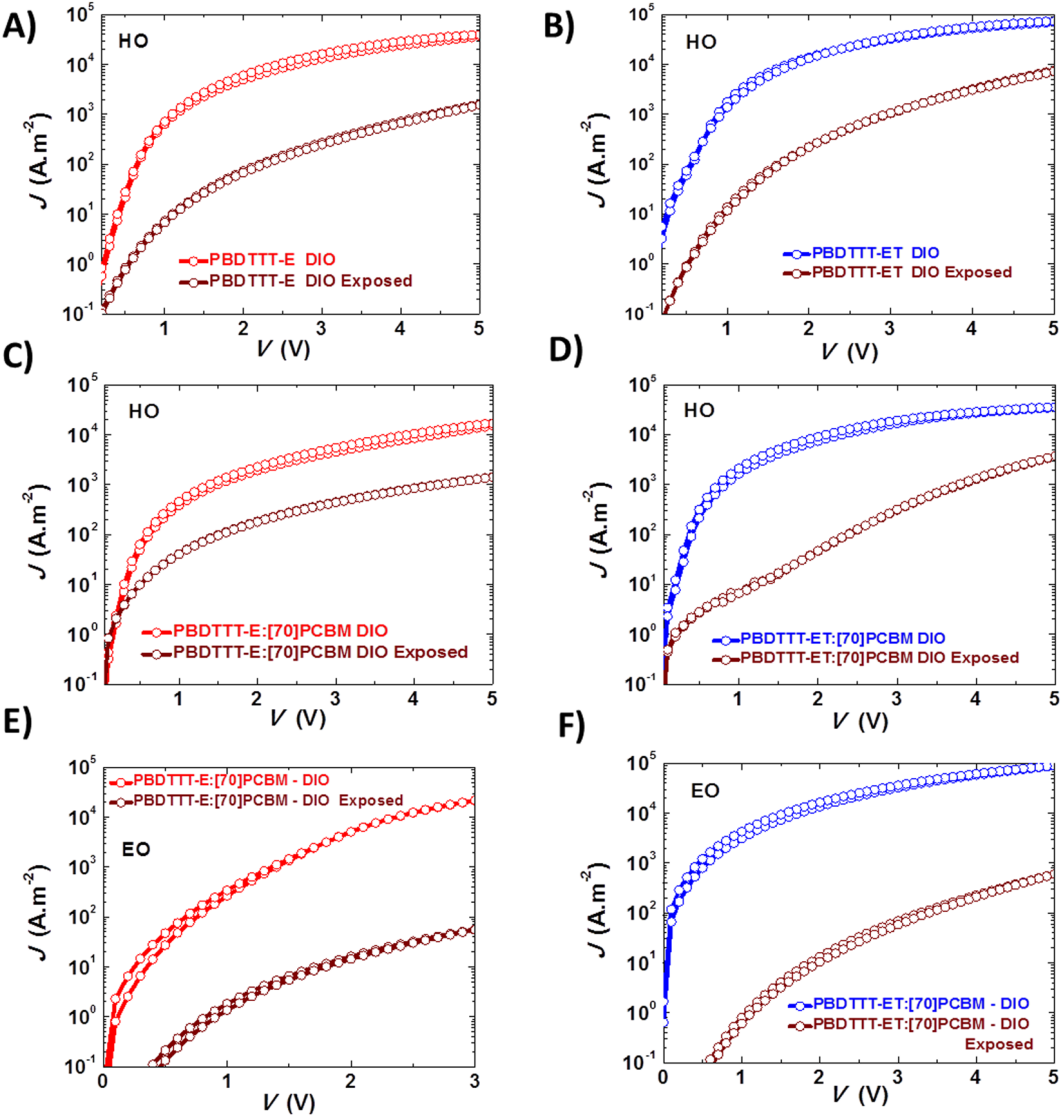
Current-voltage characteristics of unexposed (E, red and ET, blue) and exposed (1 hour - brown) of devices with DIO: Pristine polymer hole-only devices E and ET (**A**,**B**); blend hole-only devices E:[70]PCBM and ET:[70]PCBM (**C**,**D**); and blend electron-only devices E:[70]PCBM and ET:[70]PCBM (**E**,**F**).

Charge transport studies are conducted with DIO embedded films to understand the phenomenon. From the results, it seems like DIO does not affect the hole and electron currents of the fresh devices if compared to fresh devices without DIO. Next, it is apparent from Fig. 5 that in general, DIO reacts with (or affects) both polymers as well as [70]PCBM under illumination. Apart from the pronounced reduction in hole currents of the pristine polymers (Fig. 5A,B) and the electron currents of the blends (Fig. 5E,F) in similar order as observed for devices without DIO, i.e. ET > E; it is very important to single out, for instance, the huge reduction in the hole currents of the blends depicted in Fig. 5C,D. This decrease, upon illumination, is two orders of magnitude compared to the zero reduction recorded for the hole-only devices of the blends without DIO in Fig. 4C,D. This could be due to the coupled effects of DIO on both polymers and also [70]PCBM as shown in Fig. S8A in contrast to [70]PCBM without DIO in Fig. S8B. In summary, it is clear from the charge transport that ET suffered more in current reduction under illumination making ET:[70]PCBM with DIO less stable than E:[70]PCBM with DIO. This finding is perfectly in agreement with the earlier observation among the PV parameters in Fig. S4C,D, and E, that *J*_*sc*_ is the most responsible for the recorded PCE decay. Hence, DIO accelerates the degradation process but does not override the effect from the chemical structure.

To further visualize the effect of DIO in the films under UV, FTIR measurements are performed on drop cast films of polymers, fullerene, and blends from solution with DIO. For the DIO doped fullerene films in Fig. S9C, there was a continuous decrease in peaks at 1430 cm^−1^ and between 2800–3000 cm^−1^ with the disappearance of the shoulder peak at 1456 cm^−1^ and an initial increase with a subsequent decrease at the C=C stretching and at 1737 cm^−1^. In the case of the polymer films with DIO in Fig. 3C,D, first, while the peaks at the C=C and C=O stretching (1500–1570 cm^−1^ and 1714 cm^−1^) remained almost unchanged for E, a rise in these peaks is observed for ET films with DIO in contrast to ET films without DIO, leading to the appearance, at later stages of the illumination, of a carboxylic -OH peak at 3250 cm^−1^. Next, there was a large decrease (compared to films without DIO) in the peaks at the CH_2_ bending and at the aliphatic bands which was continuous for ET films but got stabilized and remained afterwards constant for E films. This is clear evidence that UV and DIO react more detrimentally with the ET polymer than the E polymer. Finally, for the blend films with DIO in Fig. S7A, B, there is a huge increase in all peaks of the fresh unexposed films except at the C=C stretching which appeared almost to be quenched in the presence of DIO but reappeared under illumination. Upon illumination, there is a big reduction in intensity of all peaks due to the combined effects of UV and DIO reactions on polymer and fullerene except for the peaks at 1714 cm^−1^ and 1737 cm^−1^ of the E blend which increased and stabilized at later stages. The substantial decrease, observed in the peaks, is continuous for ET blends but stabilises for the E blends at longer time of exposure. Similar work using FTIR to explain the mechanism of photoinduced oxidative radical reaction of PTB7-Th, PTB7-Th:[70]PCBM, and PTB7-Th:[70]PCBM with DIO has been conducted by Tremolet de Villers *et al*.^7^, with similar conclusions. Based on their analysis, they proposed a radical initiated mechanism for oxidation of PTB7-Th. They blamed the observed photoinduced oxidative reaction on the structure of PTB7-Th, pointing to the abstraction of the most acidic hydrogen, the one attached to the *α* carbon of the alkyl side chain pendant on the BDT backbone unit^7^ of PTB7-T h. In short, the FTIR data also point to the fact that upon illumination DIO is decomposed to fragments, possibly iodine radicals, that contribute to the accelerated degradation by intrinsically interacting with (the fullerene, the polymers and) the blend materials without overriding the effect of the polymer chemical structure. Similar trends and varied degree of induced degradation effects are observed for most additives, whether halogenated^7,34,35,47^ such as diiododecane, diiodohexane, diiodopentane, etc… or not^24,38–40,48^ such as octanedithiol, butanedithiol, phenylnaphthalene, chloronaphtalene, etc.

### The Role of DIO in the Degradation Explained

**Figure 6:**
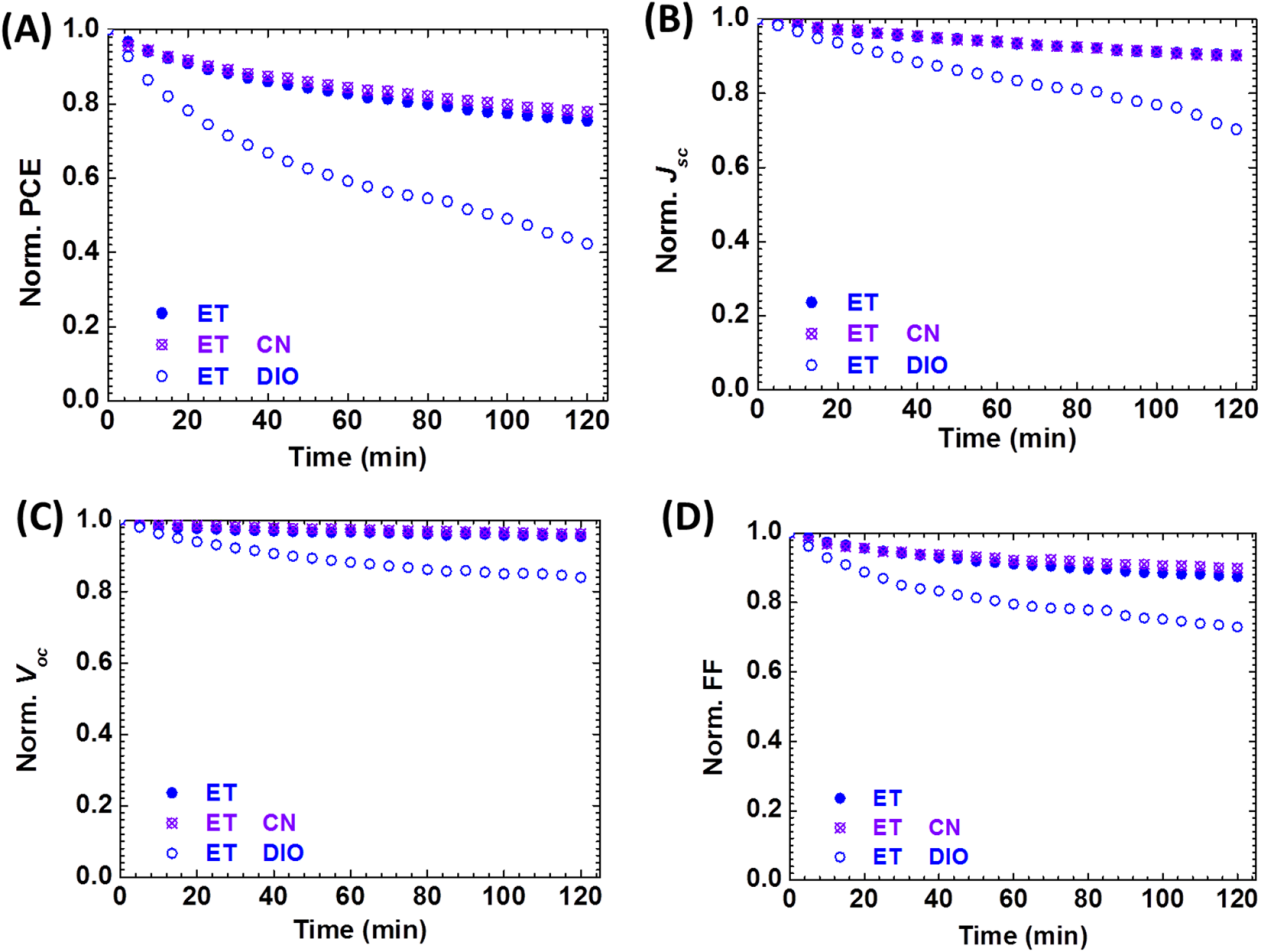
Performance of ET-based cells under continuous simulated solar illumination with no additive (blue, full symbol), with CN (purple) and with DIO (blue, empty symbol): PCE (**A**), *J*_*sc*_ (**B**), *V*_*oc*_ (**C**) and FF (**D**).

**Figure 7:**
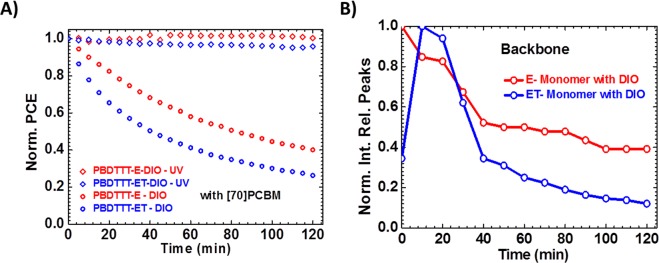
Performance of the cells with DIO, under continuous simulated solar illumination with filtered (diamond) and unfiltered (circle) lamp and ^1^H NMR integrated relative peaks to CDCl_3_ of BDT monomer solutions: Evolution of PCE of E:[70]PCBM and ET:[70]PCBM cells normalized to their initial values (**A**); and Backbone peaks evolution of E and ET BDT-monomers with DIO normalized to the highest values (**B**).

To pinpoint the exact role of DIO in the degradation process, we set out to study the DIO effect in detail to understand the mechanism. There is a possibility that DIO might have significantly altered the nanostructure morphology of the blend films of E:[70]PCBM and ET:[70]PCBM active layers under the influence of the illumination, explaining the observed pronounced degradation as opposed to the postulated iodine radicals theory; changes that were not really observable under AFM. If that were to be the case, then any light source illumination of their solar cells should cause them to degrade differently. Previously it was found that 1-chloronaphtalene (CN) precipitates the photo-oxidation (through photobleaching) of BDT-TT polymer:[70]PCBM blend films^38^. To check if CN also accelerates the photodegradation of the solar cells in our experiments, CN (≥85% technical grade with a boiling point of 260 °C) is used as an alternative to DIO. Solar cells are fabricated from blend solutions containing 0 and 3vol-% of DIO or CN and characterized under the same conditions as described above. The *J-V* parameters of these cells are displayed in Table S3. Figure 6 reveals that ET based solar cells (as well as E based cells in Fig. S9) with CN and the solar cells without DIO show the same level of stability, and in some cases, slightly better stability is observed for solar cells with CN. Only the solar cells with DIO recorded accelerated PCE decay over time. All *J-V* parameters show the same stability trends as can be seen in the figures. The fact that CN does not further degrade at all the cells (or slightly improve their stability) is offset by the observation that CN decreased the PCE of the cells as seen in Table S3. To confirm the validity of the DIO effect on all PBDT-TT polymers, PTB7, PTB7-Th, PBDTTT-C, and PBDTTT-CT are also used and similar conflicting trends of improved efficiency but accelerated photodegradation in the presence of UV-light is observed and their PCE decays are shown in Fig. S10. Figure 7A shows the combined results of devices with DIO under continuous illumination with UV-filtered and unfiltered light, leading to the conclusion that all the devices do not degrade under the UV-filtered light. First, these data reinforced the fact that the UV part of the light is responsible for the degradation. Next, not only does it reveal that UV affects the chemical structure of the polymers differently but also that it certainly reacts with the DIO molecules, through radical reaction by cleavage of carbon-iodine bonds. Thus, the formation of iodine radical species, a possible pathway of DIO reaction with UV as shown in Fig. S11, in its neutral state I_2_ or ionized states (I^−^ and I_3_^−^), can intrinsically crosslink with the polymer chemical structure, dope the film^37^, and cause pronounced detrimental reaction upon continuous UV exposure.

**Figure 8:**
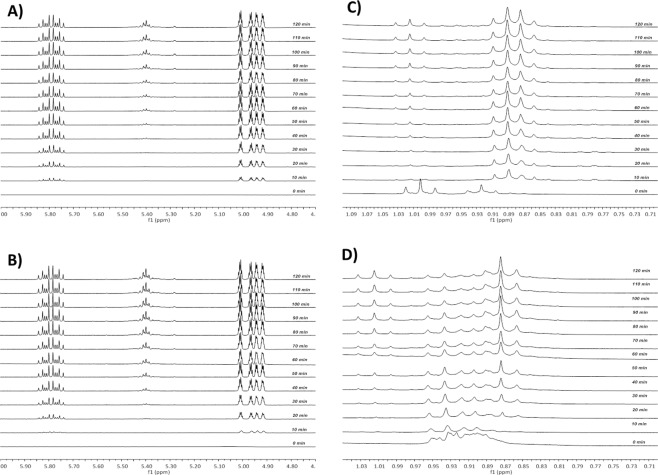
Selected peaks of 1 H NMR spectra of monomer solutions with DIO recorded in an inert environment using sealed NMR tubes under irradiation with 315–400 nm light at ten minutes intervals from bottom-to-top starting from the initial spectrum showing signals (only under illumination). Peaks between 4.8 and 6.0 ppm in E BDT- monomer (**A**), ET BDT- monomer (**B**); and 0.8 and 1.1ppm in E BDT- monomer (**C**), ET BDT- monomer (**D**). Peaks centered around 5.8 ppm belong to the vinylic proton at the 2 (or 7) position (Hc) of the alkene radical left after UV-radiation of DIO while peaks centred around 5.0 and 4.9 ppm belong respectively to the terminal vinylic protons (H_a_ and H_b_) - see Fig. 9E.

**Figure 9:**
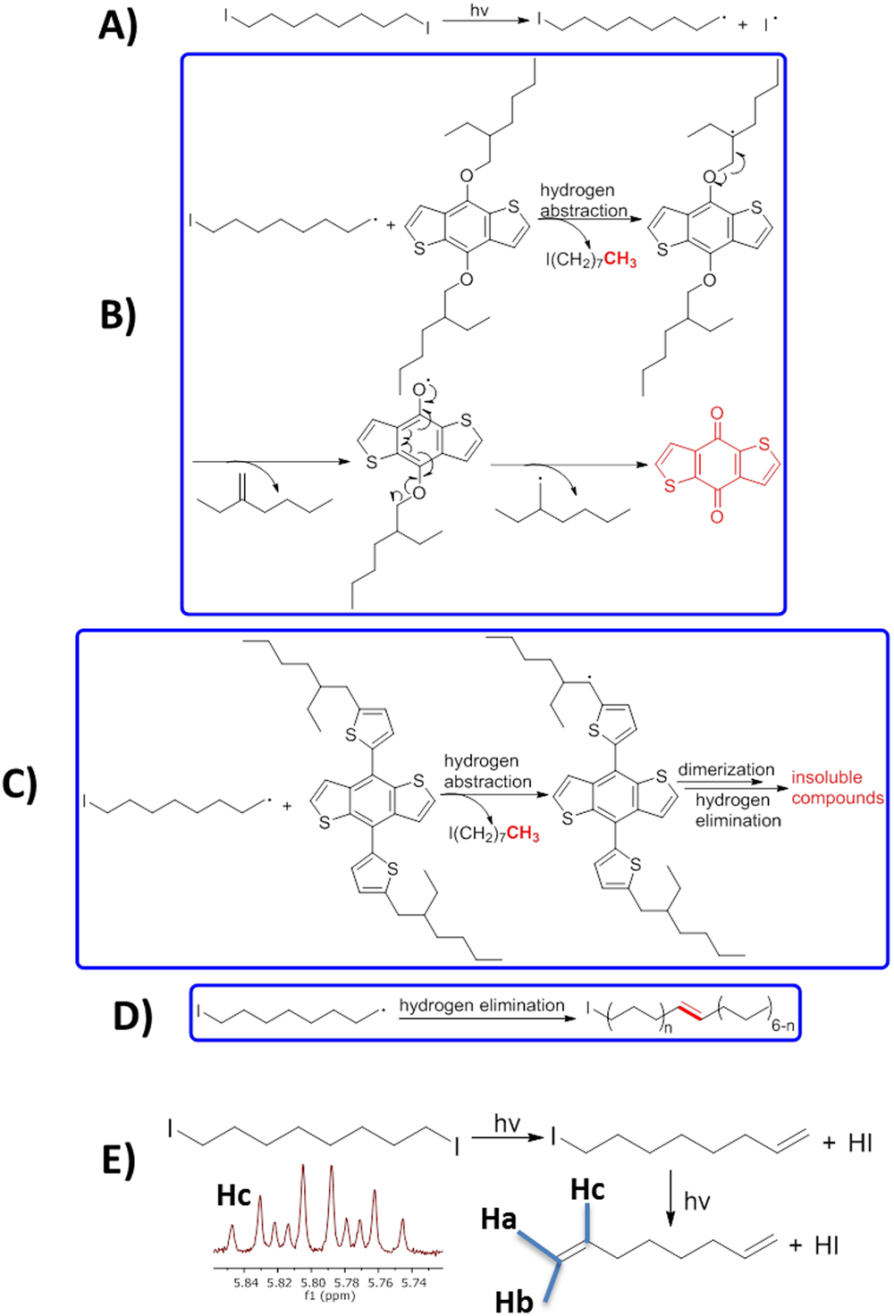
Possible degradation pathway after decomposition of DIO under UV light: Decomposition of DIO under UV light (**A**); DIO decomposition induced degradation pathways: Hydrogen abstraction in E BDT- monomer (**B**), ET BDT- monomer (**C**); and Hydrogen elimination in DIO with the formation of carbon-centered iodide radicals (**D**) and in DIO with the formation of HI (**E**), an acid supported by the ^1^H NMR splitting pattern. Shown here, H_c_ is the vinylic proton at the 2 (or 7) position (H_c_) of the alkene radical present in solution.

As earlier indicated and in previous studies^37,39,40^, DIO, a high boiling point additive could not completely be removed under high vacuum (10^−8^ Torr). This is consistent with the huge losses recorded in the mobilities of the exposed DIO films and the loss recorded in PV parameters shown in Fig. S4C,D, and E with ET recording the highest loss in *µ* and *J*_*sc*_. To explain this assertion, we first made a dilute solution of DIO-only and ODT-only (2.1 ml) with CDCl_3_. Second, we also made dilute solutions (2 ml in volume) of the BDT monomers (as shown in Fig. S12), with (0.1 ml DIO to the volume) or without of DIO, at very low concentrations. These solutions are prepared into sealed/air-tight NMR tubes in a glovebox under cleanroom environment and during measurement. Then, ^1^HNMR spectra are taken before and after UV exposure at intervals of 10 minutes for 2 hours and displayed in Fig. S13 showing the evolution of the backbone peaks of the monomers and the evolution of all other peaks for the mixture of monomers and additives and the additives-only in Figs S14–S16. The results from the NMR data partly plotted here in Fig. 7B as relative integrated peaks over time are consistent with the FTIR data. The analysis of the peaks of the backbone and the side chains revealed that the E-monomer is more stable than ET-monomer. Upon UV-exposure in the presence of DIO, there was an initial rise in peaks followed by a subsequent decrease in time. The decrease, also observed for the backbone, points to our earlier statement that the side chains upon cleavage react with the backbone, causing it to undergo slow degradation. This decrease in peaks, however, started to stabilize for the E-monomer over time while it was almost continuous with a slow rate for the ET-monomer. This observation, indeed, is clear evidence that DIO may have reacted with the polymers under UV. This reaction is undesirable for device stability in both polymer cells and even more, very detrimental to the alkylthienyl substituted polymers. Upon selection of a specific area with chemical shifts between 4.8 and 6.0 ppm of the ^1^HNMR spectra of both monomer solutions in the presence of DIO, and UV-exposure, Fig. 8A,B reveals the appearance of new peaks after 10 mins illumination that grew in intensity over time. These peaks are originally absent from the ^1^H NMR spectra of the monomer solutions without DIO. Vinyl protons are typically found in this ppm-range, suggesting the formation of alkenes during the degradation process in both monomer solutions. Peaks centered around 5.8 ppm belong to the vinylic proton at the 2 (or 7) position (H_c_) of the alkene radical left after UV-radiation of DIO while peaks centered around 5.0 and 4.9 ppm belong respectively to the terminal vinylic protons (Ha and Hb). In the aliphatic region, with chemical shifts between 0.8 and 1.1 ppm, we observed the formation of new peaks with a chemical shift around 0.87 ppm after UV-illumination as shown in Fig. 8C,D. These peaks only appeared when the solutions are exposed to UV light. From these data, we can formulate two hypotheses: either DIO reacts directly with the monomers/polymers upon UV-exposure or DIO decomposes into (radical species or other compounds which stay in) the film, in the case of the devices, altering the donor/acceptor domains and indirectly impacting the stability. These alterations could, in turn, be sources of additional electron and hole traps leading to the observed faster degradation. To clarify this point of view, we conducted a ^1^H NMR study on DIO-only and ODT-only solutions which resulted in the observation of the same peaks for the DIO-only solution, again only following UV-exposure, pointing to our second hypothesis. However, we did not see these peaks for the ODT-only solution. The fact that peaks only appear for the DIO-only solution in the olefinic and aliphatic regions of the spectra suggests that DIO undergoes homolytic C-I bond cleavage followed by hydrogen elimination to form HI and alkene as shown in Fig. 9D,E and/or followed by hydrogen abstraction. The HI, iodine and carbon-centered radicals are sufficiently reactive to react directly with the saturated carbon backbone of DIO. H^.^ abstraction^7^ has been previously speculated, though without experimental evidence, and thus being ascribed as the precursor to the phenomenon of photo-oxidative degradation of polymers in the presence of DIO when exposed to both air and light. We have shown that indeed this is partly the case isolating the molecules from oxygen in an inert atmosphere. The key finding here is that when used in the active layer of the solar cell and under illumination, DIO is a photo-acid. And this is shown for the first time using a straightforward technique as ^1^H NMR. The HI formation under the sunlight kills the cells over time. The ODT-only solution spectra revealed no changes in spectra regarding the numbers, chemical shifts and the ratio of peaks as shown in Fig. S16. Only the peak around 7.3 ppm disappeared after UV exposure, signaling an acceleration in the kinetics of deuterium exchange of the solvent with the thiol groups. These observations would explain why DIO solar cells degrade much faster in general than the ODT based and also suggest different degradation mechanisms for the additives.

Finally, based on the outcome from the NMR spectra leading to these observations, two pathways of degradation schemes were identified from DIO’s reaction (shown in Fig. 9) and could be used to explain the degradation reactions occurring in the active layers: H^.^ abstraction induced degradation and H^.^ elimination induced degradation. Figure 9A shows that upon UV illumination, DIO first goes through homolytic cleavage to produce a primary alkyl radical and iodine radical. Because of the high reactivity of the primary alkyl radical, it readily abstracts hydrogen from the E-monomer, which gives iodooctane with a terminal methyl group (which appears around 0.87 ppm) and a relatively stable tertiary alkyl radical, followed by the homolytic cleavage of the C-O bond to form more stable oxygen radical and an alkene. The occurrence of a homolytic cleavage of the other C-O bond and rearrangement of the radical leads to the formation of a stable quinone moiety in Fig. 9B along with a primary alkyl radical, propagating further decomposition. Thus, in the presence of DIO, there are two competing/cumulative routes to quinone formation: one due to DIO reaction with the polymer and the second due to a direct UV-reaction^32^ with the polymer. This process explains why DIO accelerates the degradation process in E-monomer and thus the E-polymer solar cells. In the case of the ET-monomer, as shown in Fig. 9C, a secondary alkyl radical which is stabilized by the thiophene instead of a third alkyl radical is produced by the hydrogen abstraction reaction. Although this radical is relatively stable, dimerization and hydrogen elimination can still occur due to the reactivity of the radical, leading to the production of insoluble compounds not observable from the ^1^H NMR spectra. This reaction mechanism also strongly supports the fact that DIO indeed speeds up the degradation of ET-monomer and thus, ET-polymer solar cells. Finally, and as shown in Fig. 9D, hydrogen elimination of DIO itself also occurs, forming HI which is a strong acid, and competing simultaneously with the other pathways. HI is highly reactive and would kill the cells. The formation of HI generates alkenes in the system, which is evident from the ^1^H NMR spectra arising from the vinylic protons shown in Fig. 9E. This observation is further supported by the predicted ^1^H NMR spectrum of the same alkene chain as depicted in Fig. S17. It should be noted that in general, the same photochemistry is probably active, over a long period, in some other additives. Alkane dithiols^48^, for example, are contaminated with disulfides that are difficult to remove completely, form readily upon exposure to ambient conditions and cleave homolytically in the presence of UV light.

## Conclusion

We have furnished evidence that reveals that BDT-TT polymers are intrinsically unstable in the presence of UV-light illumination if compared to the known fairly stable P3HT. We have shown that in general, polymers with ether groups on the BDT-unit are more stable than those with thiophene groups when exposed continuously to full solar spectrum. Thus, UV reacts more negatively with the BDT substituted thiophene group polymers, creating radical species that accelerate the degradation mechanism during the first step of their degradation pathway compared to that of the ether group polymers. These observations have been supported by the charge transport studies, FTIR, ^1^H-NMR measurements and UV absorption profiles of the monomer solutions. In brief, these findings corroborate the proposition that polymer chemical structure plays a crucial role in the photostability of polymer-fullerene solar cells.

We have also confirmed that the addition of DIO precipitates the UV-degradation and thus, negatively affects the photostability, reducing the lifetime of the cells, on the one hand from more than 2 hours to 20 minutes for the E polymer cells and on the other hand from 30 minutes to less than 10 minutes for the ET polymer cells. However, it does not alter/tweak the influence of the polymer chemical structure; thus, the photostability of BDT-TT polymer solar cells is strongly linked to the polymer structure. We moved a step further to explain the mechanism behind the observed precipitation in degradation of the cells caused by the combined effect of the addition of the DIO and UV-exposure; and henceforth propose schemes of these mechanisms, supported by experimental evidence, for the differences observed for both types of polymers. All this information lead to two simple conclusions:

- BDT-TT polymers are unstable, and their chemical structure is a key factor in the performances of their polymer solar cells, thus, in our case, PBDTTT-E is more photostable than PBDTTT-ET

- DIO and other (mostly halogenated) additives become photo-acids and are inimical to device stability as it is challenging to completely remove their residues from the film with 100% certainty.

These findings inform us of ways to achieve efficient but stable materials and additives.

## Methods

### Materials and Processing

#### Materials

The E and ET polymers and [70]PCBM were purchased from Solarmer Energy Inc. and Solenne BV respectively, while P3HT and all used solvents are acquired from Sigma-Aldrich Co. LLB. The BDT monomers are synthesized in our laboratory as described and shown previously^32^. All commercial materials are used as received.

#### Solution Processing

For the solar cells: (i) blend of P3HT polymer with [60]PCBM (in a ratio of 1:1 with a total concentration of 20 mg.ml^−1^) were dissolved in anhydrous CB and (ii) blends of either E polymer with [70]PCBM (or ET polymer in a ratio of 1:1.5 with a total concentration of 25 mg.ml^−1^) were dissolved in anhydrous oDCB with or without DIO. For single carrier devices, either [70]PCBM (60 mg.ml^−1^) or pristine polymers (20 mg.ml^−1^) or blends as described above are similarly dissolved. When DIO is added, it was in a volume ratio of 97:3 for oDCB:DIO. The solutions were mostly dissolved overnight by stirring on a hot plate at 60 °C.

### Device and Film Fabrication

Glass or pre-patterned ITO glass substrates were cleaned respectively in soap, deionized water, then in acetone and isopropanol with ultrasonic bath for at least 10 mins each and spin-dried. Next, the substrates were annealed in an oven at 140 °C for 10 mins and then treated in UV-Ozone for 20 mins. The dissolved materials were used to either fabricate conventional solar cells or single carrier devices or films on glass. For solar cells, the blend solutions were spin coated at 800 rpm for 5 s and spin dried for 120 s atop PEDOT:PSS (VP AI4083, H.C. Starck) layer (50 nm, and dried in oven at 140 °C for 10 mins), previously spin coated in ambient conditions on the pre-patterned ITO glass substrate. The films were left in vacuum overnight, and the devices were finished by thermal evaporation at <10^−8^ Torr of LiF (1 nm) and Al (100 nm) with the following structure ITO/PEDOT:PSS/Blend with or without DIO/LiF/Al. The P3HT solar cells were annealed at 150 °C for 30 minutes. For single carrier devices the solutions were spin coated at 600 rpm for 5 s and spin dried for 120 s atop their respective substrates with electron-only devices having the following structure Al (20 nm)/[70]PCBM (or Blend) with or without DIO/LiF/Al and hole-only devices having the following structure Cr (1 nm)/Au (20 nm)/Pristine polymer (or Blend) with or without DIO/Pd (15 nm)/Au (80 nm). The thickness of the active layers of the solar cells is around 100 nm while that of the single carrier devices is 115 nm for the blend layers, 100 nm for the pristine polymer layers and 130 nm for [70]PCBM layers. Finally, films of pristine polymers, of [70]PCBM and of blends were made either by spin-coating on glass substrates for AFM and UV-Vis absorption measurements or by drop casting on KBr crystals for FTIR measurements.

### Characterization

#### J-V characteristics, UV-Vis Absorption, and ^1^H NMR

*J-V* characteristics of the solar cells and the single carrier devices and UV-Vis Absorption measurements are taken as previously described^32^. The light source for the efficiency and degradation measurement is a SolarConstant 1200 Steuernaugel metal halide lamp calibrated to 1 sun intensity and corrected for the spectral mismatch with the AM1.5 G spectrum using a Si reference cell. Blend films on glass or diluted solutions of the BDT monomers of E and ET in CDCl_3_ at very low concentration were prepared in an inert atmosphere in a glovebox in sealed cuvettes (0.7 ml in volume) for the absorption measurements. Additionally, diluted solutions of the BDT monomers of E and ET in CDCl_3_ at very low concentration are also prepared in an inert atmosphere in a glovebox, namely, two times 2.1 ml (in 95.2:4.8 v/v ratio of CDCl_3_:DIO) and 2 ml without DIO in air-tight/sealed NMR tubes for the NMR measurements. To complete the NMR measurements, 2.1 ml diluted solutions of DIO-only and ODT-only in CDCl_3_ (95.2:4.8 v/v ratio of CDCl_3_:DIO/ODT) are prepared under the same condition. Finally, absorption measurements were performed similarly to that of the films in a UV-3600 Shimadzu UV-Vis-NIR spectrometer against a cuvette of CDCl_3_ as a reference.

#### FTIR Measurement

The measurements were performed on both as the cast and exposed films against KBr crystal as a reference with a Shimadzu IR-Tracer-100 FTIR spectrometer in transmittance mode from 400 to 4000 cm^−1^.

#### AFM measurement

Exposed and as-cast blend films of E:[70]PCBM and ET:[70]PCBM on glass are investigated for morphological differences in ScanAsyst mode on a Bruker Multimode 8 microscope (Model number: MMAFM-2) with ScanAsyst-Air probes (spring constant: 0.4 N/m, resonant frequency: 70 kHz, nominal tip radius: 2 nm). All samples are scanned at 5 µm, 1 µm, and 500 nm at a scan rate of 0.8 Hz and a resolution of 640 samples per line. Both height and peak force errors are collected for analysis during the scan. We used NanoScope Analysis (provided by Bruker) for data analysis and converting 2D morphology into the 3D structure for better interpretation.

## Supplementary information

Supplementary Info_DIO acts as a photoacid in organic solar cells
