# Supplementary material for: 1,8-diiodooctane acts as a photo-acid in organic solar cells
Source: Sci Rep. 2019 Mar 13;9:4350. doi: 10.1038/s41598-019-40948-1 (PMC6416288; doi:10.1038/s41598-019-40948-1)
Supplement: Supplementary file 1 — Supplementary Info_DIO acts as a photoacid in organic solar cells [file 41598_2019_40948_MOESM1_ESM.pdf]

Supplementary Information

1,8-diiodooctane acts as a photo-acid in organic solar cells

Nutifafa Y. Doumon,[a,*] Gongbao Wang,[b] Xinkai Qiu,[a,b] Adriaan J. Minnaard,[b] Ryan C. Chiechi[a,b] and L. Jan Anton Koster[a,*]

[a] *PhotoPhysics and OptoElectronics, Zernike Institute for Advanced Materials, University of Groningen, Nijenborgh 4, NL-9747 AG, Groningen-The Netherlands*

[b] *Stratingh Institute for Chemistry, University of Groningen, Nijenborgh 4, NL-9747 AG, Groningen-The Netherlands*

**n.y.doumon@rug.nl*, *l.j.a.koster@rug.nl*

1. Device counts and optical properties of pristine and blend materials

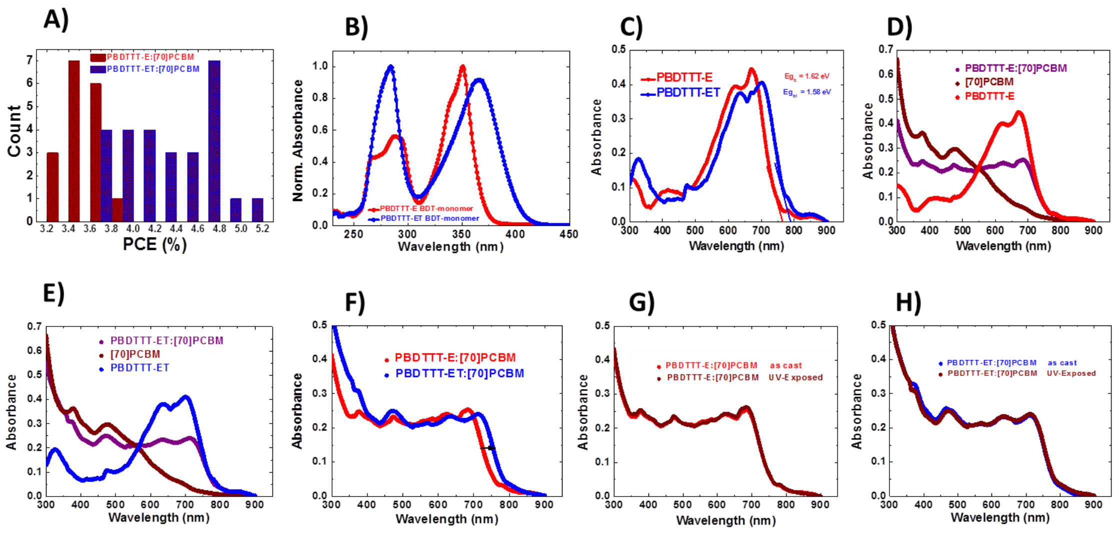

Figure S1. Devices and Optical properties. (A) Device count vs. PCE for E:[70]PCBM and ET:[70]PCBM solar cells; UV-Vis absorption spectra of (B,C) BDT-monomers and polymers (D,E) fullerene, polymers and blends (F) the two blends showing the red-shifted onset of ET and (G,H) the two blends before and after UV-exposure.

2. AFM images of blend films

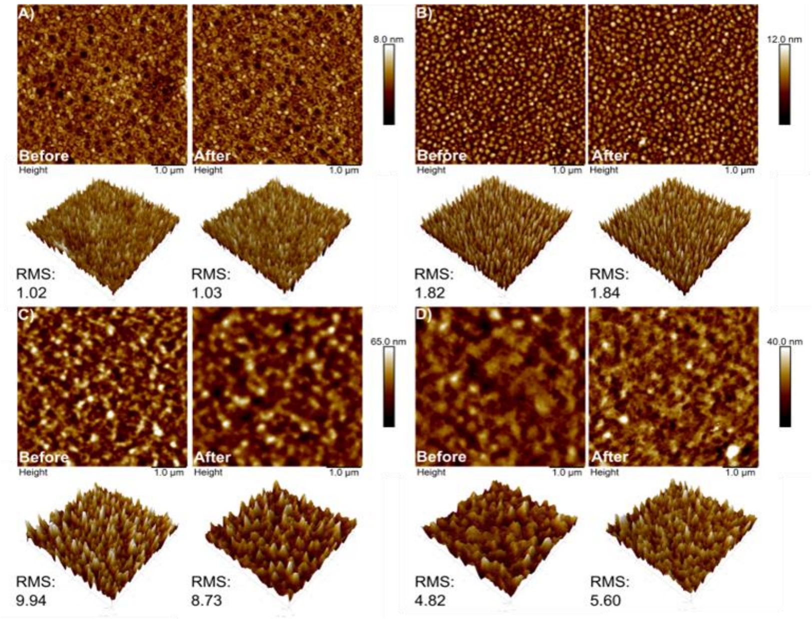

Figure S3. Height profiles of spin-coated films without vacuum treatment before and after solar simulator lamp exposure (2 hrs) with a scan size of 5 um. For each section, top half shows the morphology of the film before/after UV exposure, the bottom half shows the 3D topography of the film before/after UV exposure. (A,B) Films E:[70]PCBM and ET:[70]PCBM without DIO (C,D) Films of E:[70]PCBM and ET:[70]PCBM with DIO.

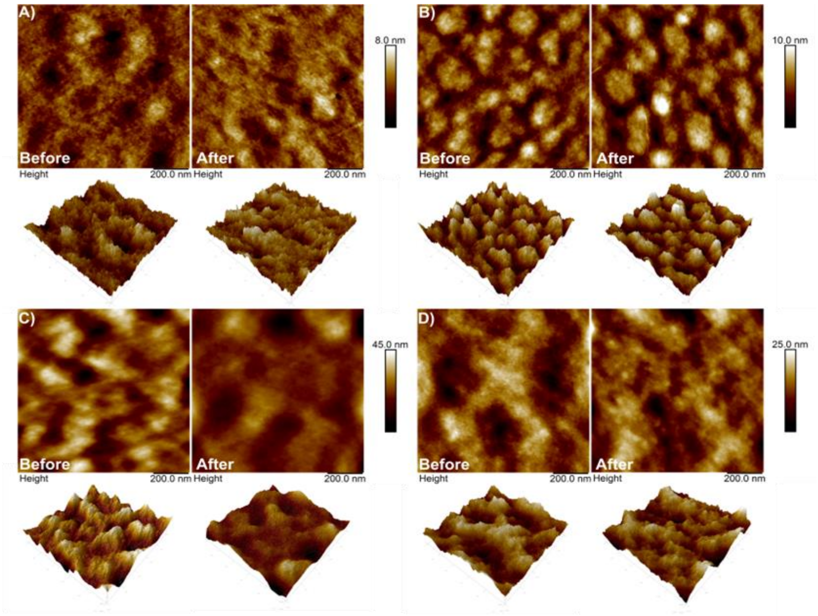

Figure S4. Height profiles of spin-coated films without vacuum treatment before and after solar simulator lamp exposure (2 hrs) with a scan size of 1 um. For each section, top half shows the morphology of the film before/after UV exposure, the bottom half shows the 3D topography of the film before/after UV exposure. (A,B) Films E:[70]PCBM and ET:[70]PCBM without DIO (C,D) Films of E:[70]PCBM and ET:[70]PCBM with DIO.

3. Degradation of the two blends solar cells under filtered and unfiltered lamp

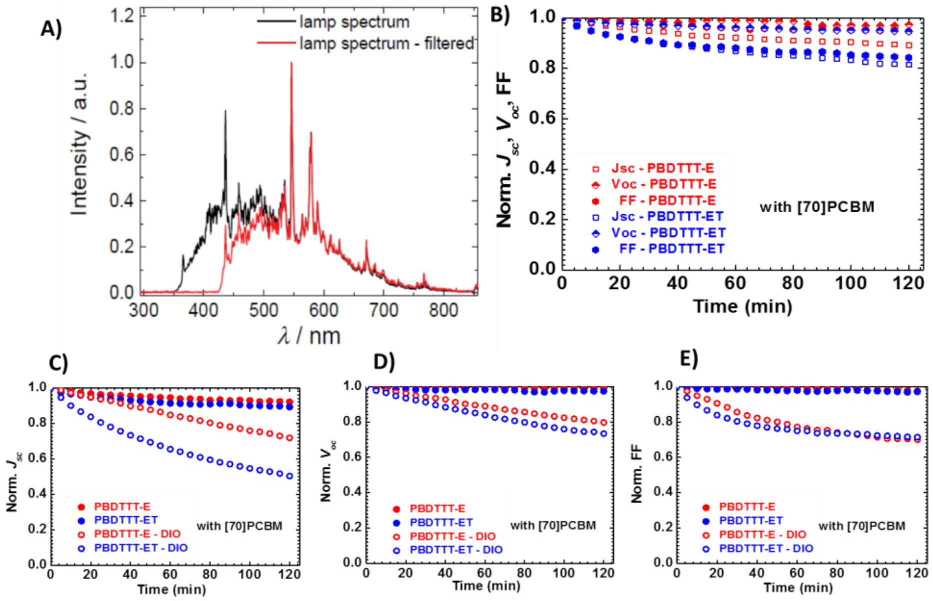

Figure S5. (A) Spectra oft he light oft he solar simulator recorded with and without the Long pass filter in front of the spectrometer,[1] (B) Evolution of J_{sc}, V_{oc}, and FF of E:[70]PCBM and ET:[70]PCBM cells without DIO under unfiltered lamp normalised to their initial values. Performance under continuous simulated solar illumination of the cells with (Full symbols) and without DIO (Empty symbols): evolution of (C) J_{sc}, (D) V_{oc}, and (E) FF of E:[70]PCBM and ET:[70]PCBM cells.

4. Charge transport in pristine polymer and blend single carrier devices

Table S1. Mobilities (μ) of unexposed (E, red and ET, blue) and exposed (brown) films of single or double carrier devices obtained by space charge limited current (SCLC) measurements.

Polymer	Solvents	Treatment	μ_h Pristine 10^{-4} ($cm^2.V^{-1}.s^{-1}$)	μ_h Blend 10^{-4} ($cm^2.V^{-1}.s^{-1}$)	μ_e Blend 10^{-4} ($cm^2.V^{-1}.s^{-1}$)
PBDTTT-E	oDCB	as cast	8.5	4	7
		Exposed	4.5	4	2.5
PBDTTT-E	oDCB:DIO	as cast	8	1.1	4
		Exposed	0.09	0.095	0.025
PBDTTT-ET	oDCB	as cast	19	5	12
		Exposed	3.1	5	0.5
PBDTTT-ET	oDCB:DIO	as cast	55	4.5	90
		Exposed	0.35	0.018	0.020

Holes (h) and electrons (e)

The obtained data from the charge transport measurements are fitted with modified Mott–Gurney equation:[1]

$$J = \frac{9}{8}\,\varepsilon_o \varepsilon_r \mu_{on} \exp(0.891\gamma_n \sqrt{\frac{V_{int}}{L}})\,\frac{V_{int}^2}{L^3} \qquad (1)$$

where J is SCLC density, ε_o and ε_r are the electric permittivity of free space and the relative dielectric constant of the active layer respectively, μ_{on} is the charge carrier mobility, L is the thickness of the device and γ_n is the electric field-activation factor, with the voltage on the active layer given by:

$$\boldsymbol{V_{int} = V - V_{bi} - V_{rs}} \qquad \textbf{(2)}$$

where V is the applied voltage, V_{bi} the built-in voltage and V_{rs} is the voltage drop due to the series resistance of the contacts.

5. FTIR of pristine and blend films

Table S2. FTIR Absorption band assignments for [70]PCBM (black), E and E:[70]PCBM (red), ET and ET:[70]PCBM (Blue) films.

Assignments	Pristine E / γ (cm^{-1})	Pristine ET / γ (cm^{-1})	[70]PCBM / γ (cm^{-1})
CH_3 Symmetric Bending	1365	1378	1329
CH_2 Bending	1460	1460	1456 & 1430
(Thieno) thiophene ring/band	943	943	
(C=C stretching)	1500-1565	1514-1570	1480-1584
Carbonyl Group (C=O stretching)	1714	1714	1737
Aliphatic (hydrogen) bands	2750-3000	2750-3000	2800-3100
CH_2 Symmetric Stretching	2867	2859	
CH_2 Asymmetric Stretching	2930	2925	
CH_2 Asymmetric Stretching	2956	2959	
-CH Stretching	3080	3074	
Hydroxy (-OH) Groups	3100-3500	3100-3500	
Carboxylic -OH	-	3210*	
Alcoholic -OH	-	3405	

* Observed only in the presence of DIO

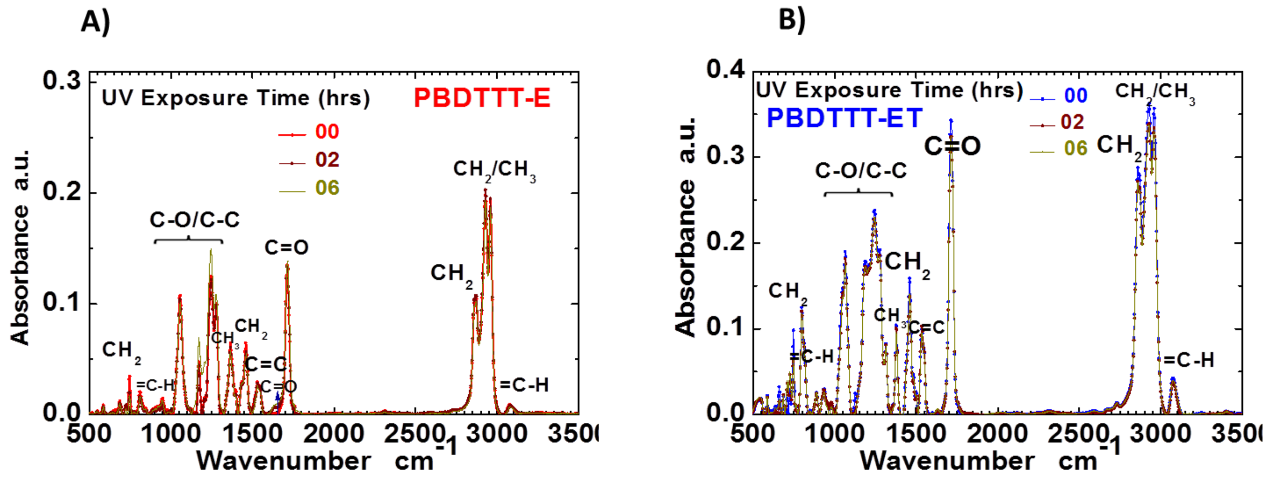

Figure S5. Full FTIR absorption spectra of unexposed and exposed (2 hours, 4 hours and 6 hours) films of pristine and blend materials without DIO: (A) E-polymer and (B) ET-polymer.

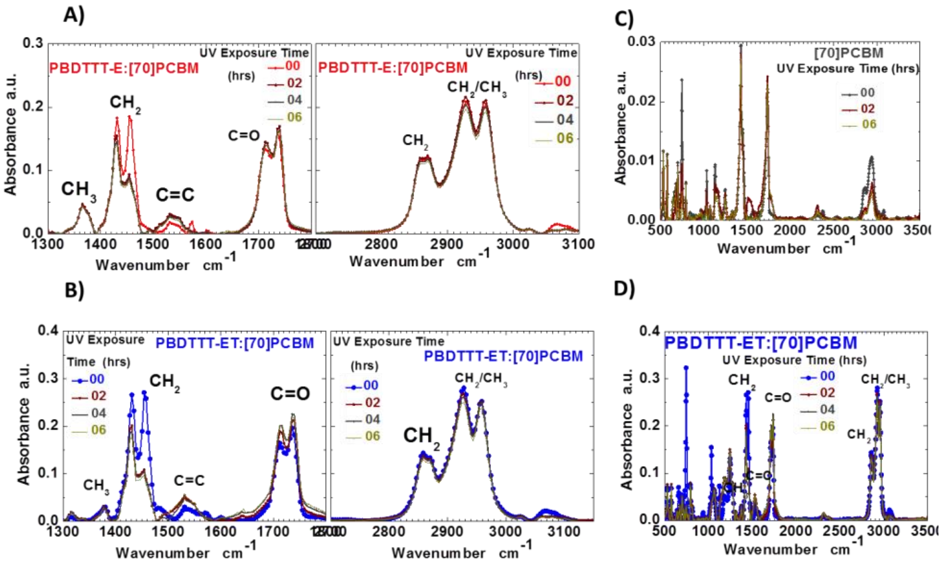

Figure S6. (Full) FTIR absorption spectra of unexposed and exposed (2 hours, 4 hours and 6 hours) films of pristine and blend materials without DIO: (A) E:[70]PCBM blend, (B) ET:[70]PCBM blend, (C) [70]PCBM, and (D) ET:[70]PCBM blend.

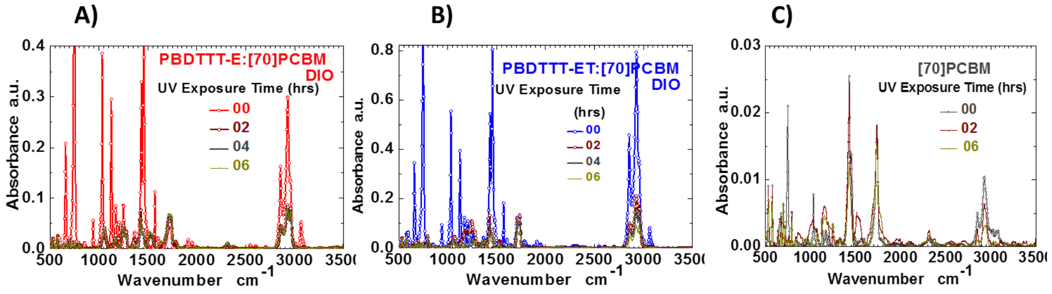

Figure S7. Full FTIR absorption spectra of unexposed and exposed (2 hours, 4 hours and 6 hours) films of pristine and blend materials with DIO. (A) E:[70]PCBM blend, (B) ET:[70]PCBM blend, and (C) [70]PCBM.

6. Charge transport in unexposed and exposed [70]PCBM films

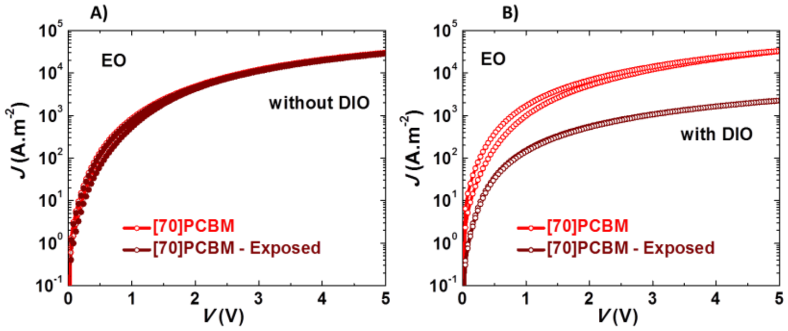

Figure S8. Current-voltage characteristics of unexposed (red) and exposed (1 hour - brown) of [70]PCBM devices (A) without DIO and (B) with DIO.

7. Devices with different additives and different polymers

Table S3. Device parameters of solar cells processed from oDCB, oDCB:CN and from oDCB:DIO.

Polymer/Solvents	L (nm)	J_{sc} ($A.m^{-2}$)	V_{oc} (V)	FF (%)	PCE (%)
PBDTTT-E/oDCB	100	97	0.671	56.7	3.7
PBDTTT-E/oDCB:CN	100	87	0.660	56.4	3.2
PBDTTT-E/oDCB:DIO	100	118	0.611	62.7	4.5
PBDTTT-ET/oDCB	100	115	0.717	56.3	4.7
PBDTTT-ET/oDCB:CN	100	103	0.740	56.3	4.3
PBDTTT-ET/oDCB:DIO	100	113	0.688	63.2	4.9

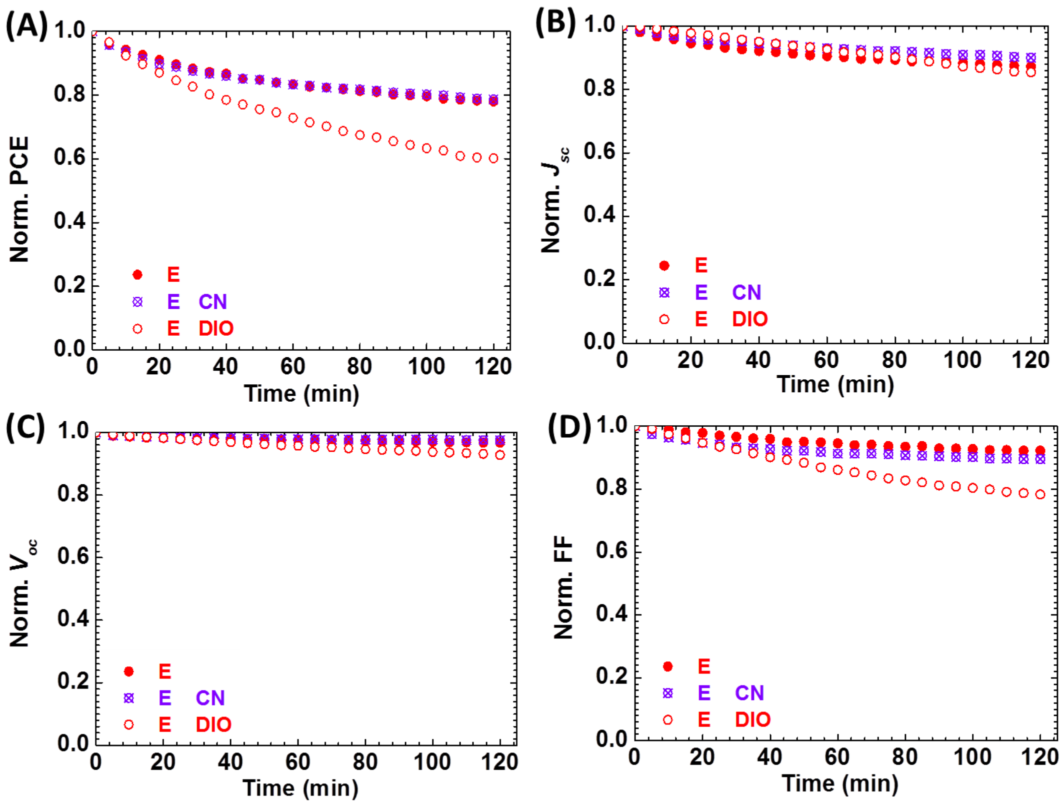

Figure S9. Performance of E based cells under continuous simulated solar illumination with no additive (red, full symbol), with CN (purple) and with DIO (red, empty symbol): PCE (A), J_{sc} (B), V_{oc} (C) and FF (D).

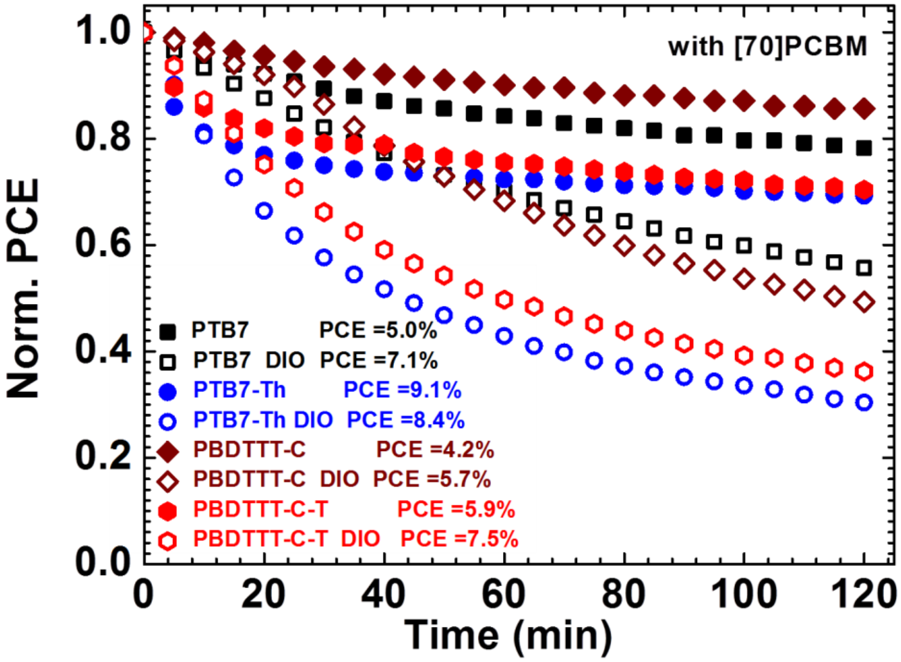

Figure S10. PCE performance of other PBDT-TT polymer-based cells over time under continuous simulated solar illumination without DIO (full symbol) and with DIO (empty symbol).

8. DIO reaction pathways, Monomers & Integrated ^{1}HNMR Spectra

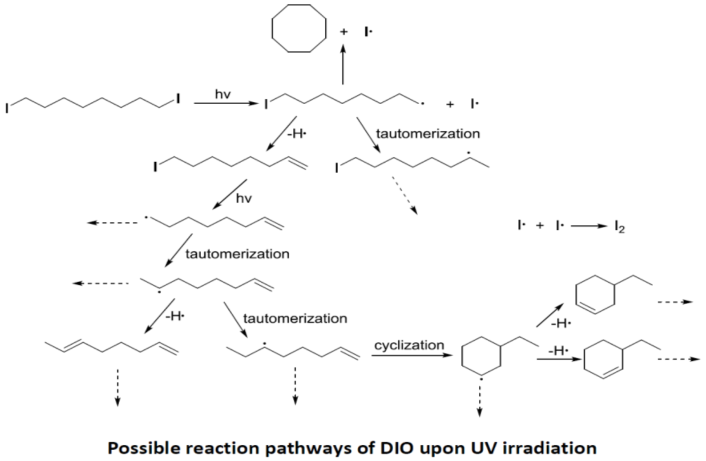

Figure S11. Reaction pathways of DIO upon UV-radiation

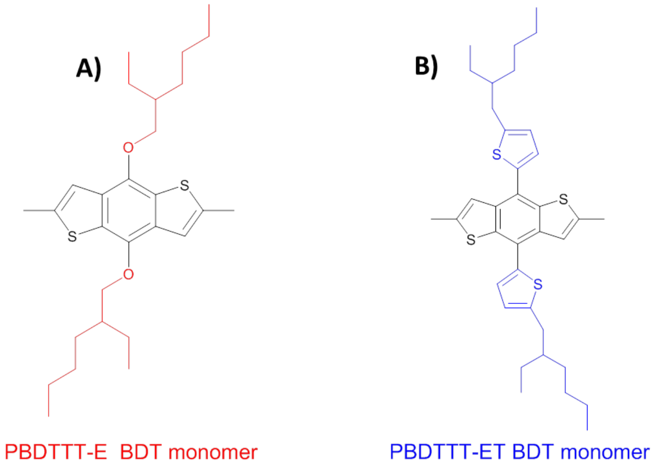

Figure S12. Chemical structure of (A) E BDT-monomer and (B) ET BDT-monomer used for ^{1}H-NMR and absorption spectra.

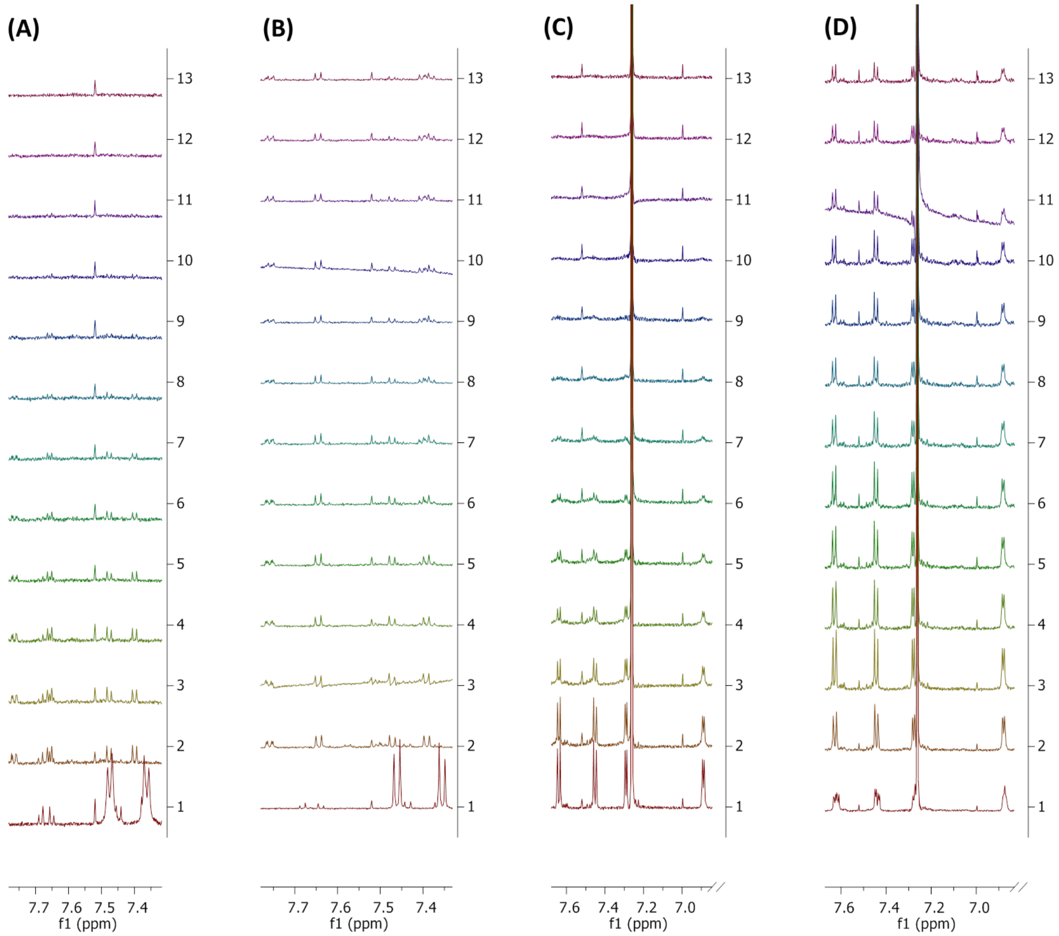

Figure S13. ^{1}HNMR spectra of monomer solutions showing the backbone peaks recorded in an inert environment using sealed NMR tubes under irradiation with 315-400 nm light at ten minutes intervals from bottom-to-top starting from the initial spectrum labelled as 1. E BDT-monomer (A) without DIO and (B) with DIO; ET BDT- monomer (C) without DIO and (D) with DIO.

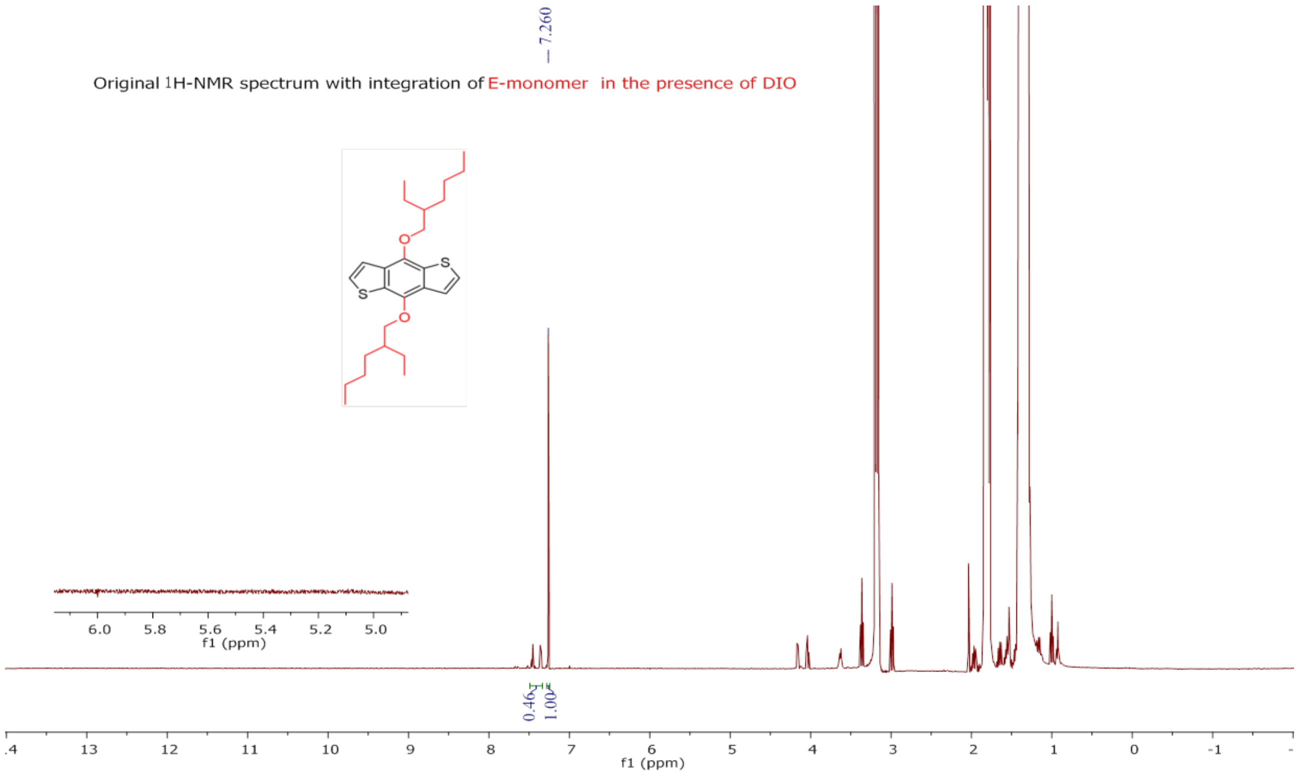

Original 1H-NMR spectrum with integration of E-monomer in the presence of DIO
7.260
0.46
1.00
f1 (ppm)

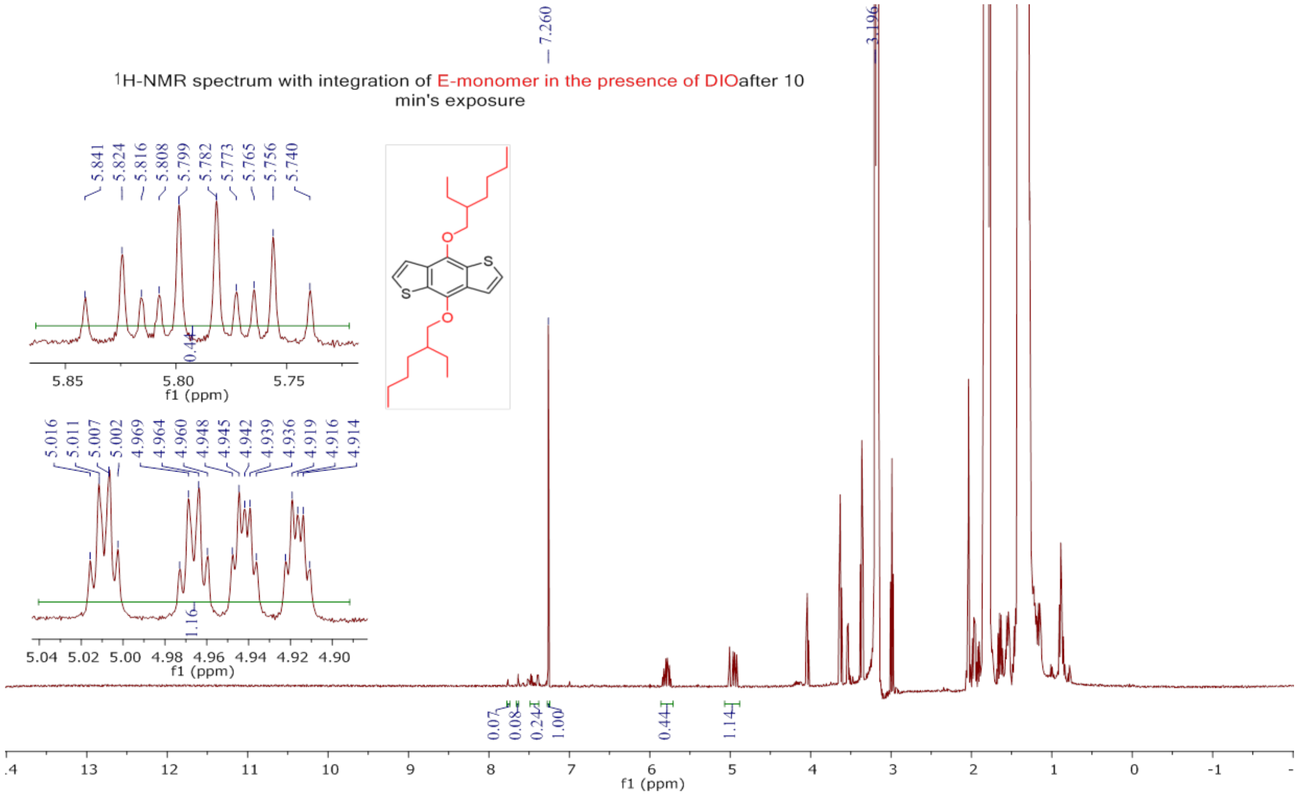

1H-NMR spectrum with integration of E-monomer in the presence of DIOafter 10 min's exposure
7.260
3.196
5.841
5.824
5.816
5.808
5.799
5.782
5.773
5.765
5.756
5.740
0.44
5.016
5.011
5.007
5.002
4.969
4.964
4.960
4.948
4.945
4.942
4.939
4.936
4.919
4.916
4.914
1.16
0.07
0.08
0.24
1.00
0.44
1.14
f1 (ppm)

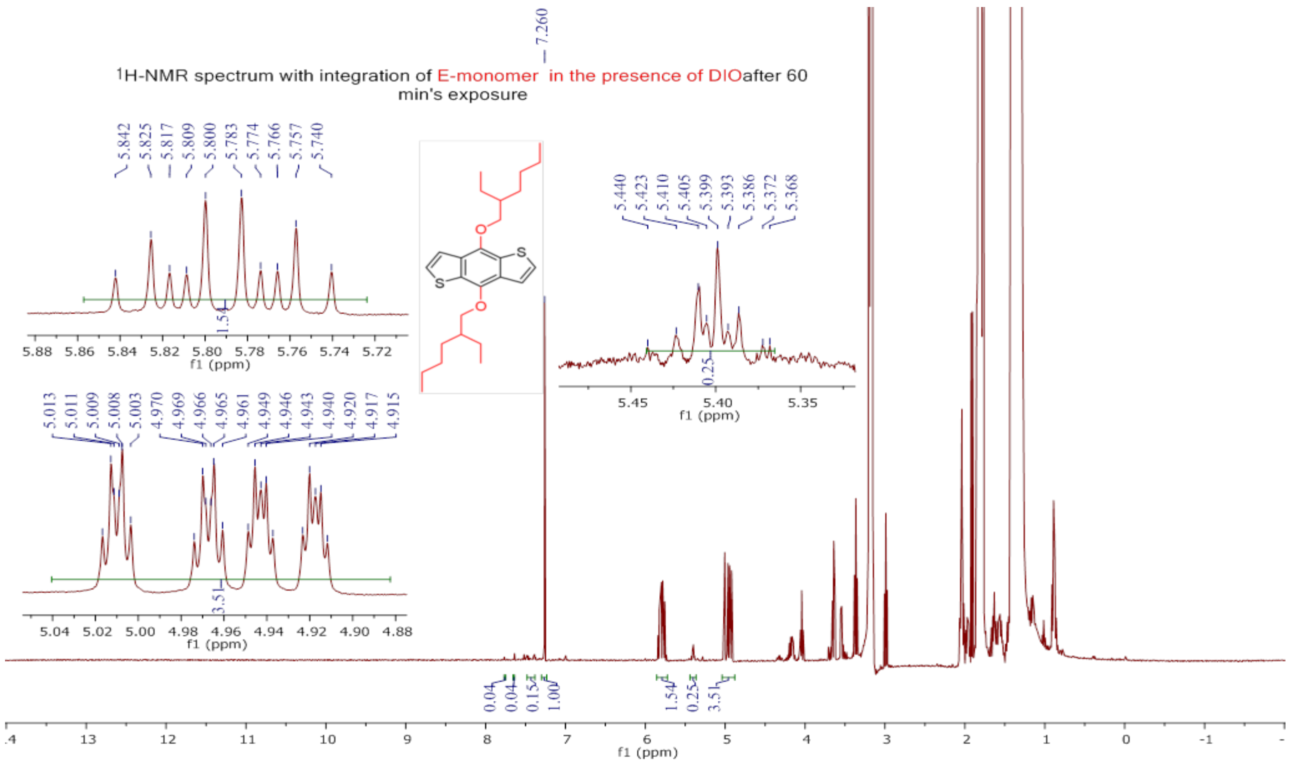

1H-NMR spectrum with integration of E-monomer in the presence of DIOafter 60 min's exposure
f1 (ppm)

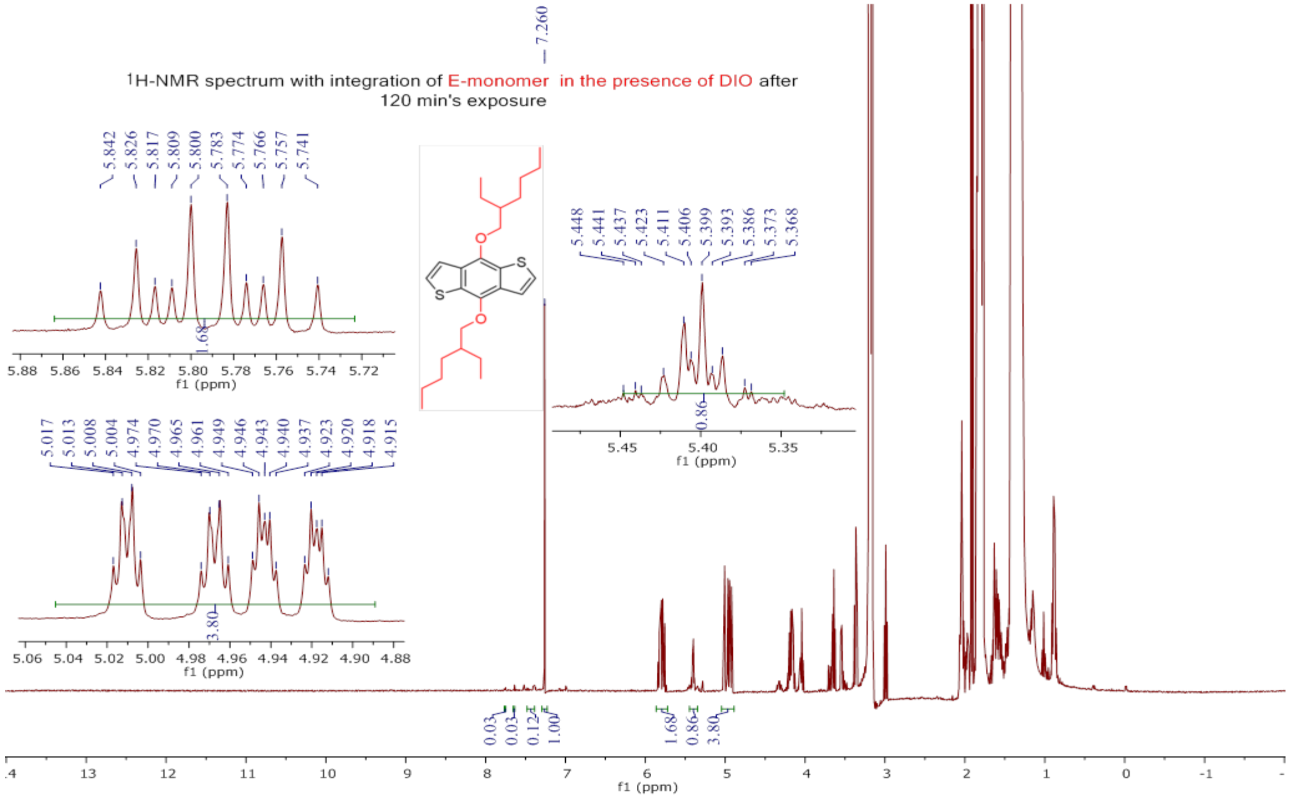

1H-NMR spectrum with integration of E-monomer in the presence of DIO after 120 min's exposure
f1 (ppm)

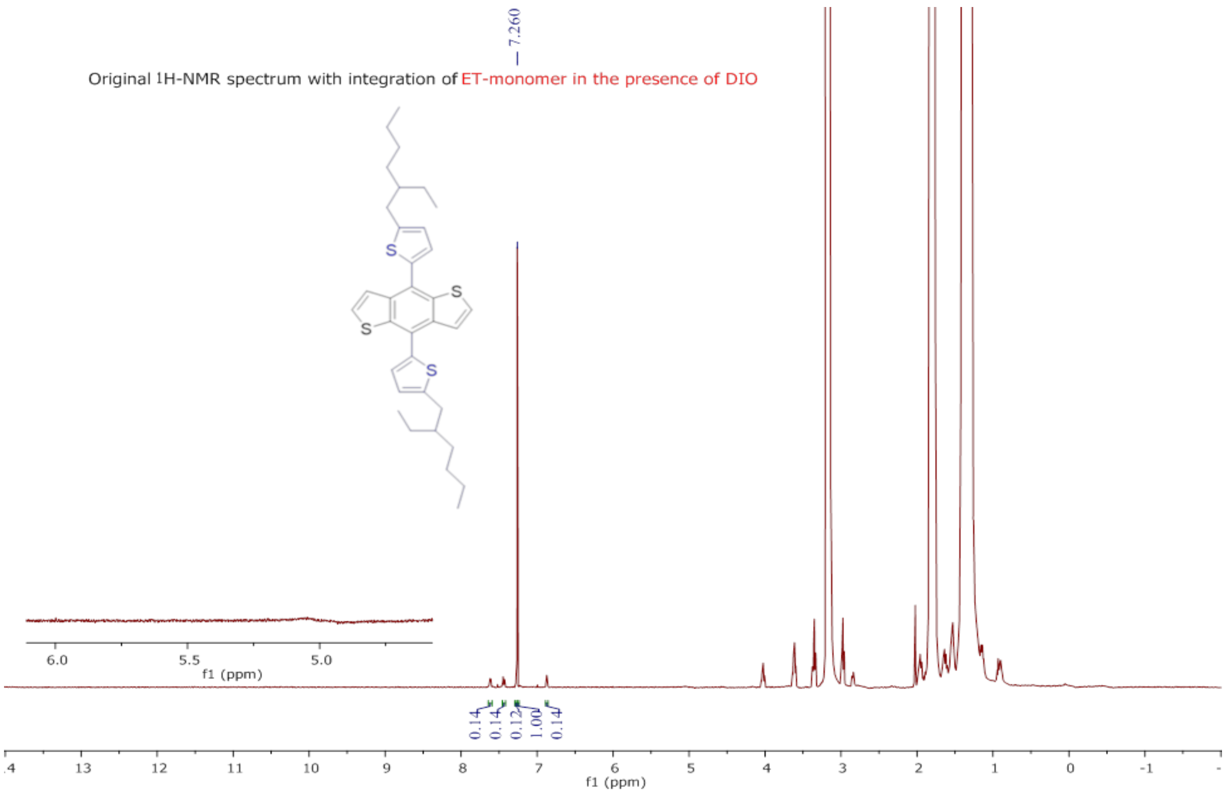
Original 1H-NMR spectrum with integration of ET-monomer in the presence of DIO
7.260
S
S
S
S
6.0
5.5
5.0
f1 (ppm)
0.14
0.14
0.12
1.00
0.14
13
12
11
10
9
8
7
6
5
4
3
2
1
0
-1
f1 (ppm)

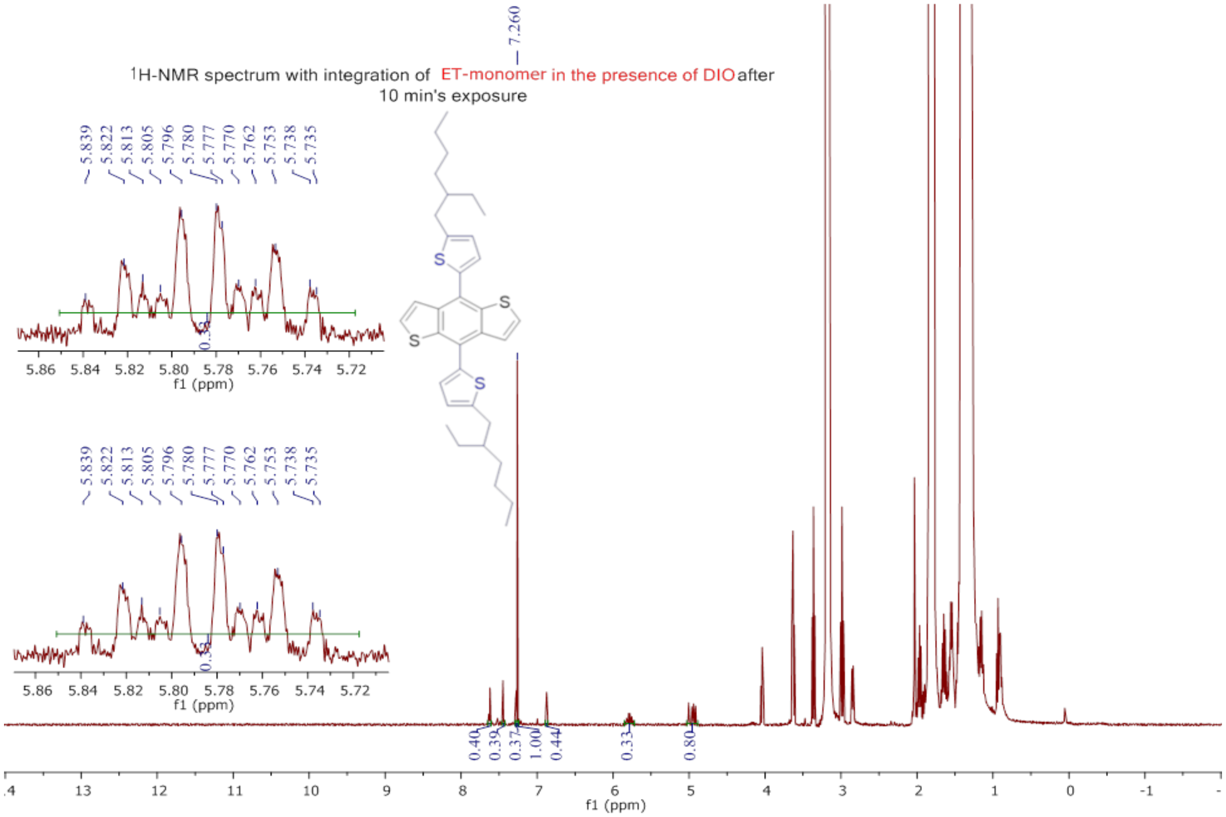
1H-NMR spectrum with integration of ET-monomer in the presence of DIO after 10 min's exposure
7.260
5.839
5.822
5.813
5.805
5.796
5.780
5.777
5.770
5.762
5.753
5.738
5.735
5.86
5.84
5.82
5.80
5.78
5.76
5.74
5.72
f1 (ppm)
S
S
S
S
0.40
0.39
0.37
1.00
0.44
0.33
0.80
13
12
11
10
9
8
7
6
5
4
3
2
1
0
-1
f1 (ppm)

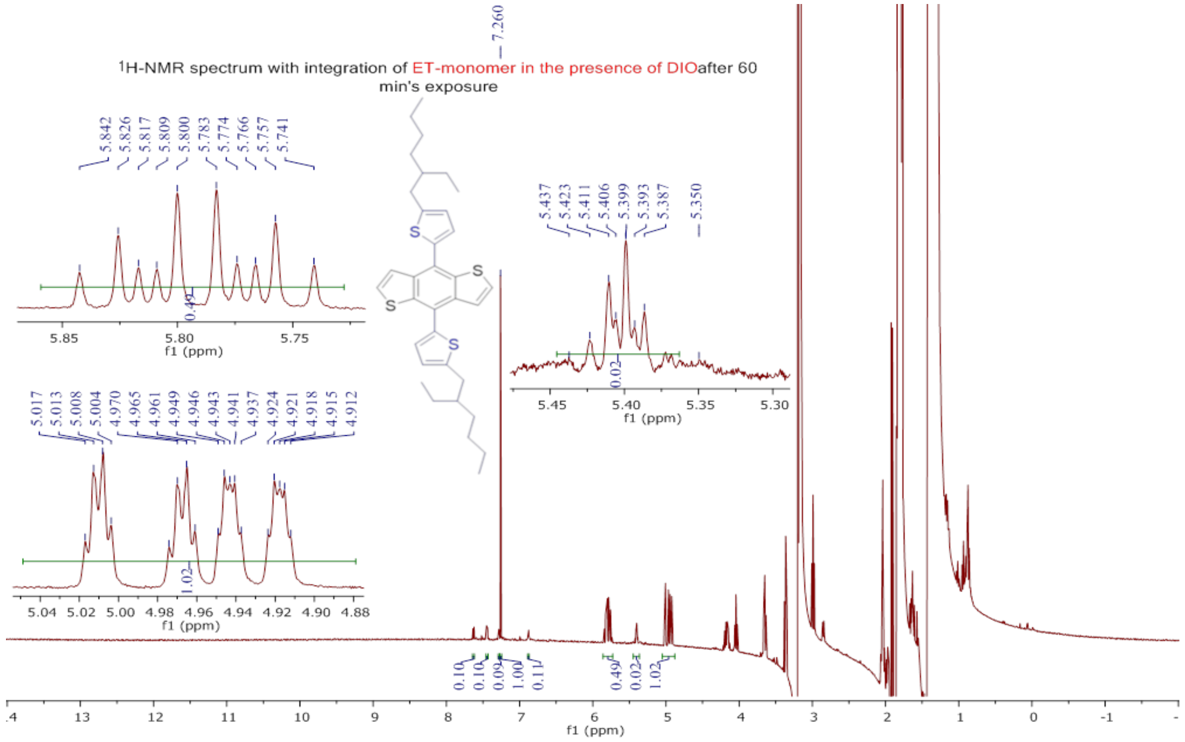

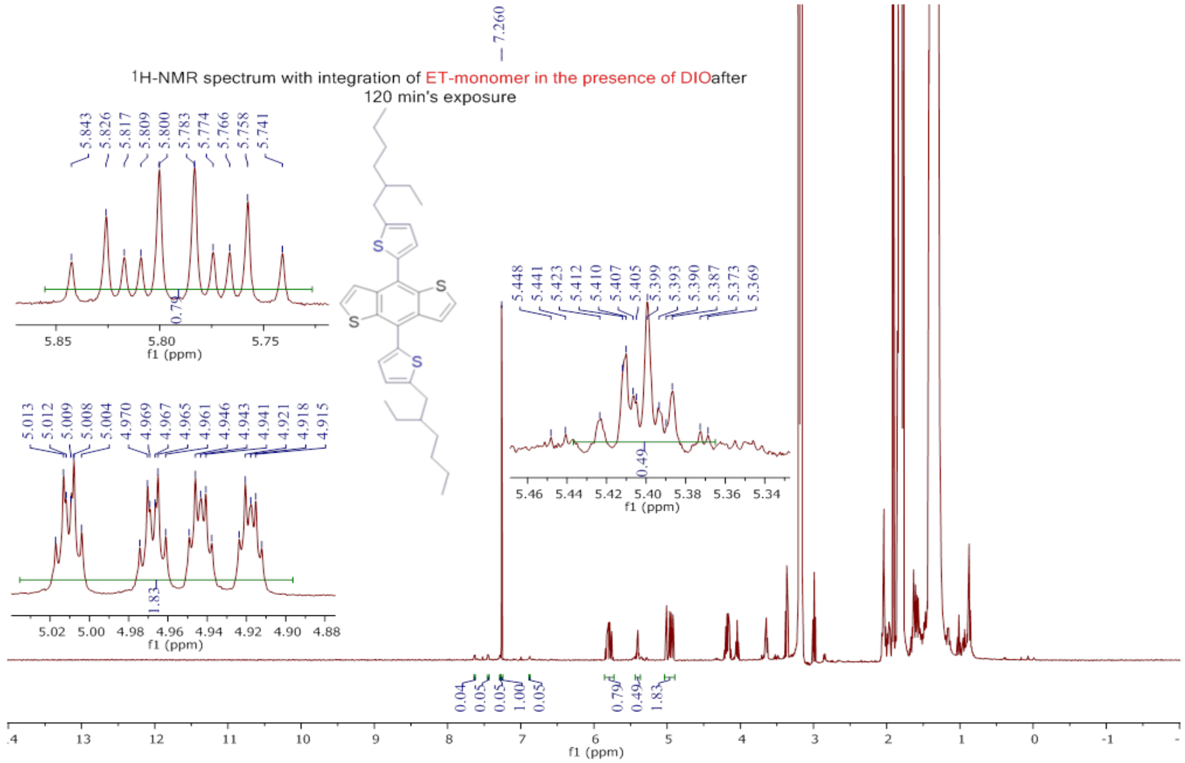

Figure S14. Integrated ^{1}HNMR spectra of monomer solutions with DIO recorded in an inert environment using sealed NMR tubes under irradiation with 315-400 nm light at ten minutes intervals showing only the initial spectrum and spectra after 10 and 120 mins.

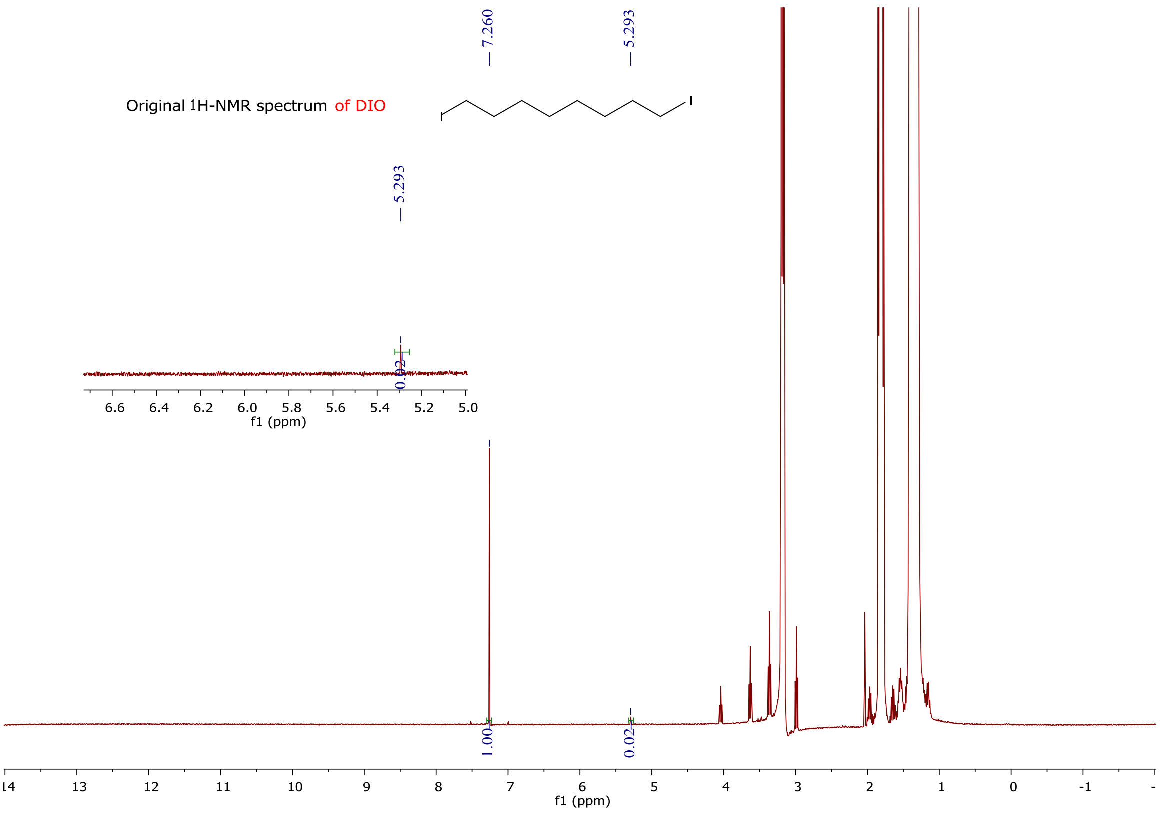
Original 1H-NMR spectrum of DIO
I
I
7.260
5.293
1.00
0.02
f1 (ppm)

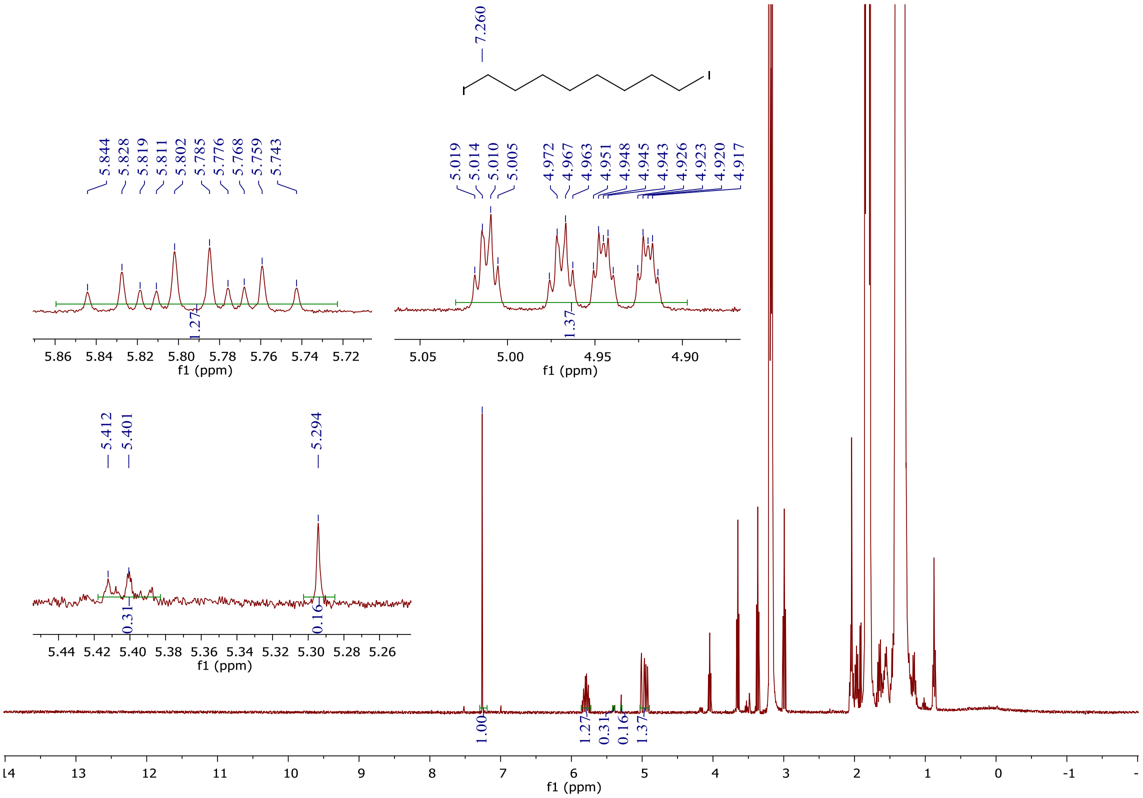
I
I
7.260
5.844
5.828
5.819
5.811
5.802
5.785
5.776
5.768
5.759
5.743
5.019
5.014
5.010
5.005
4.972
4.967
4.963
4.951
4.948
4.945
4.943
4.926
4.923
4.920
4.917
5.412
5.401
5.294
1.00
1.27
0.31
0.16
1.37
f1 (ppm)

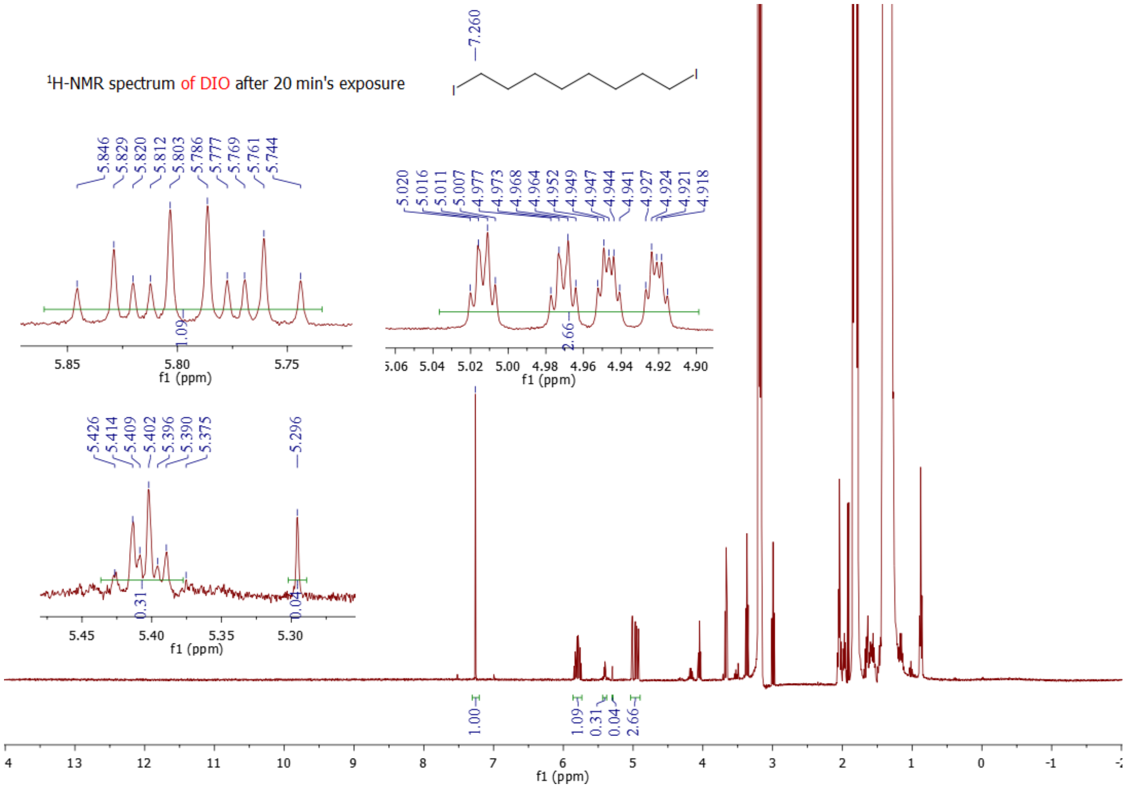

1H-NMR spectrum of DIO after 20 min's exposure

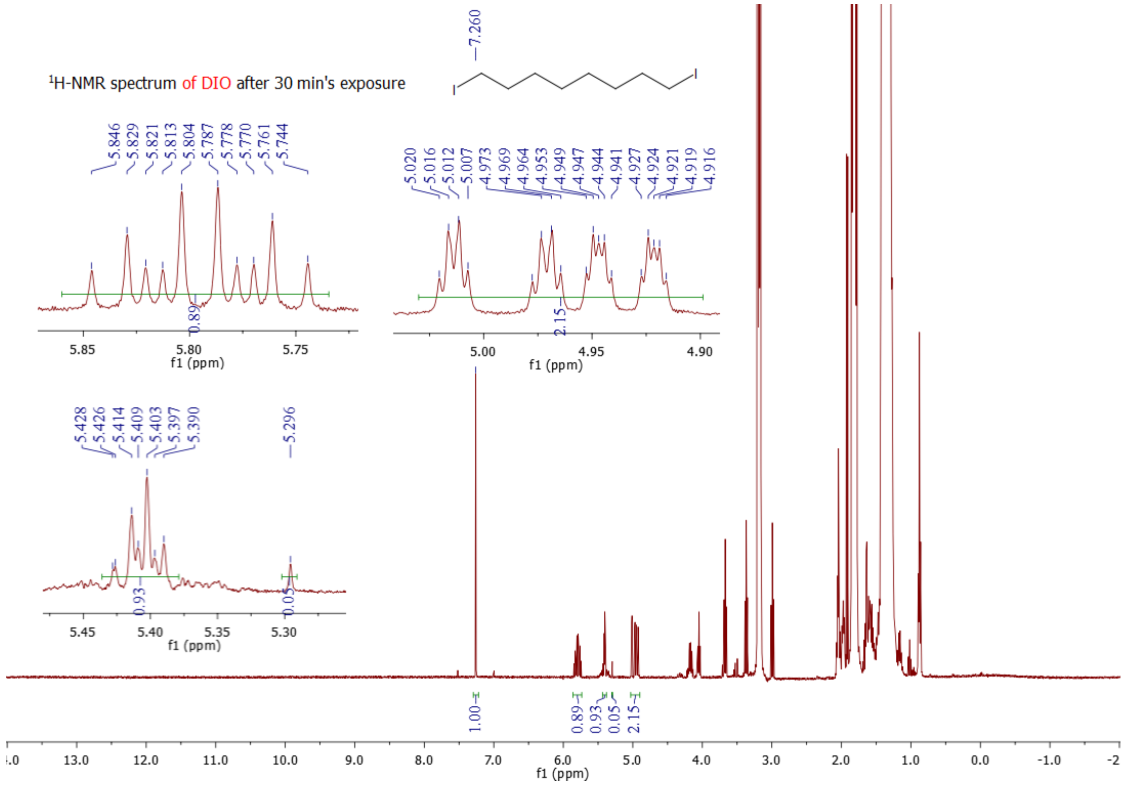

1H-NMR spectrum of DIO after 30 min's exposure

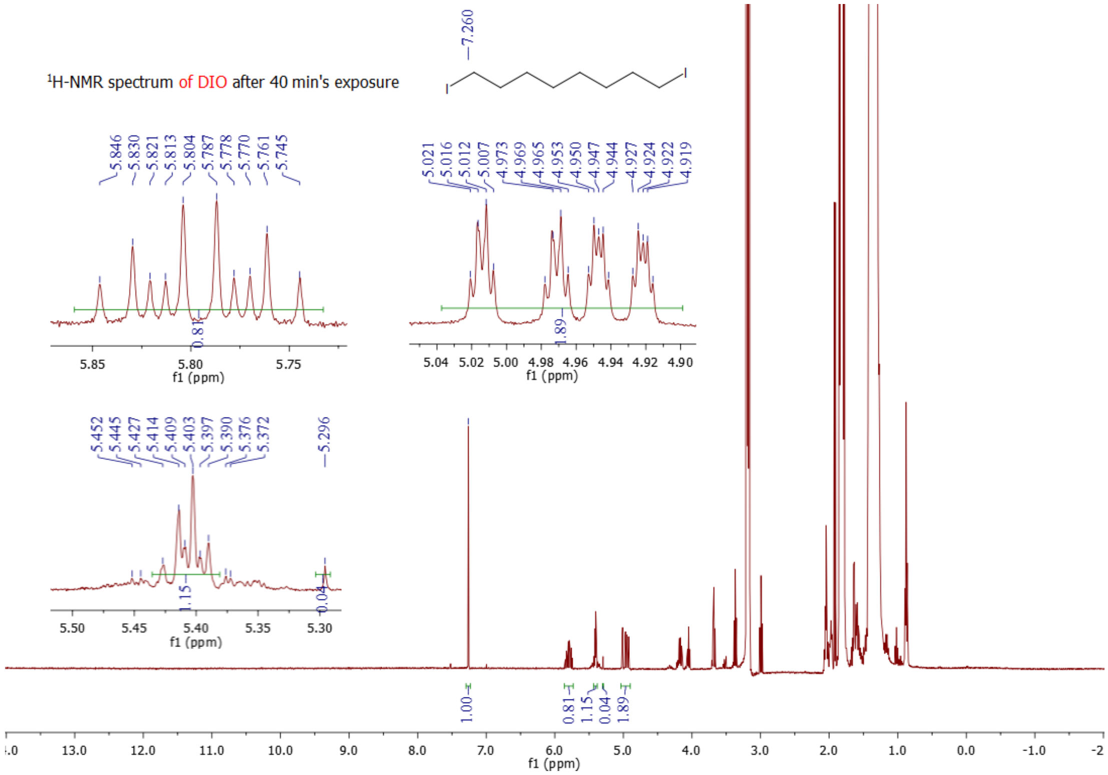

¹H-NMR spectrum of DIO after 40 min's exposure
f1 (ppm)

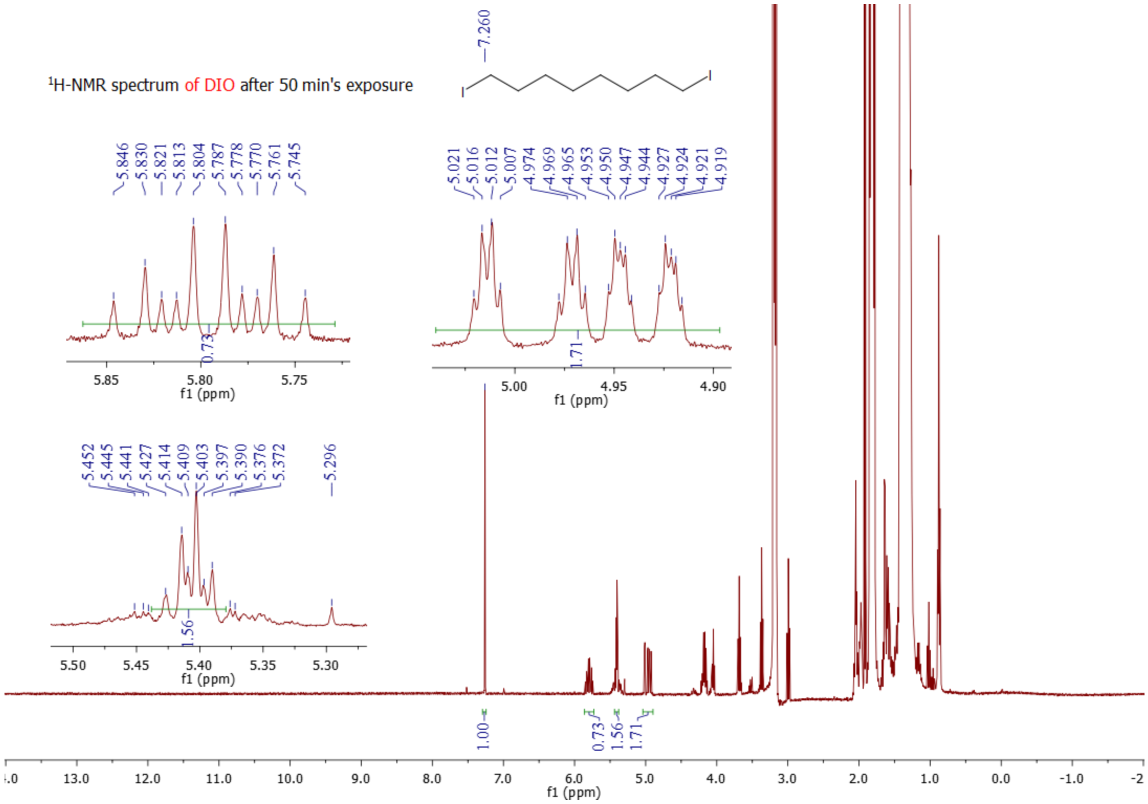

¹H-NMR spectrum of DIO after 50 min's exposure
f1 (ppm)

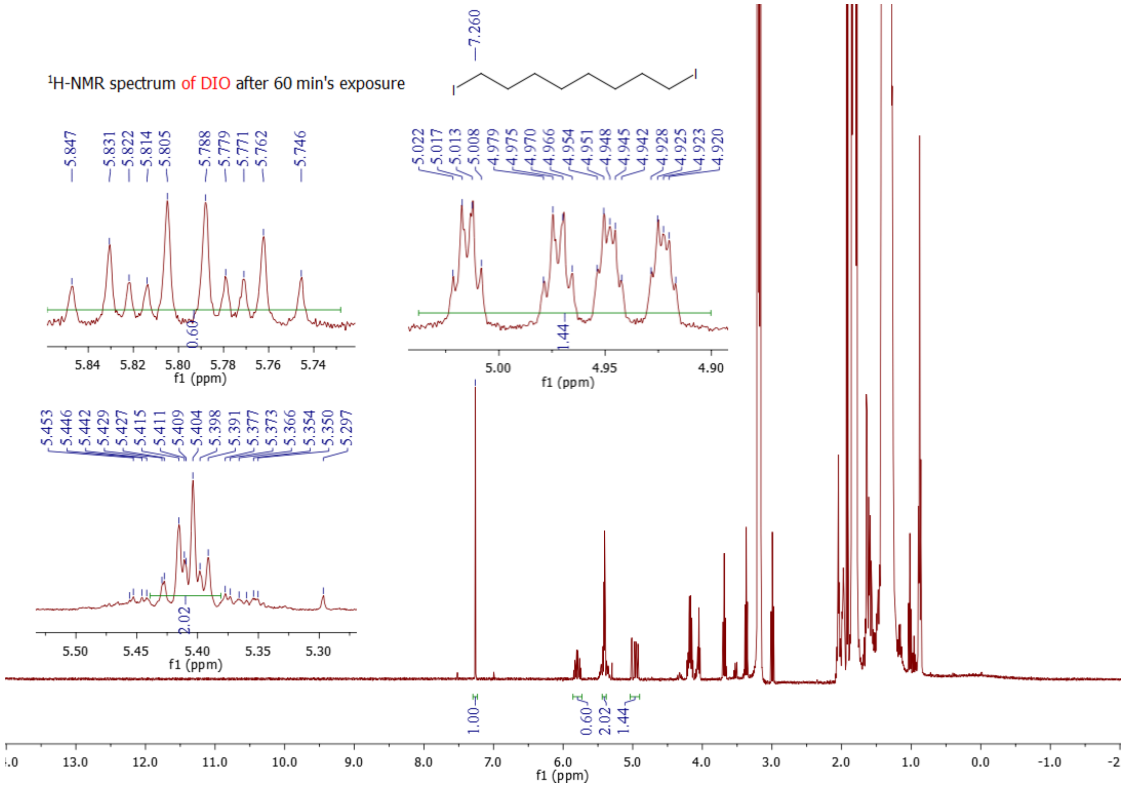

1H-NMR spectrum of DIO after 60 min's exposure
f1 (ppm)

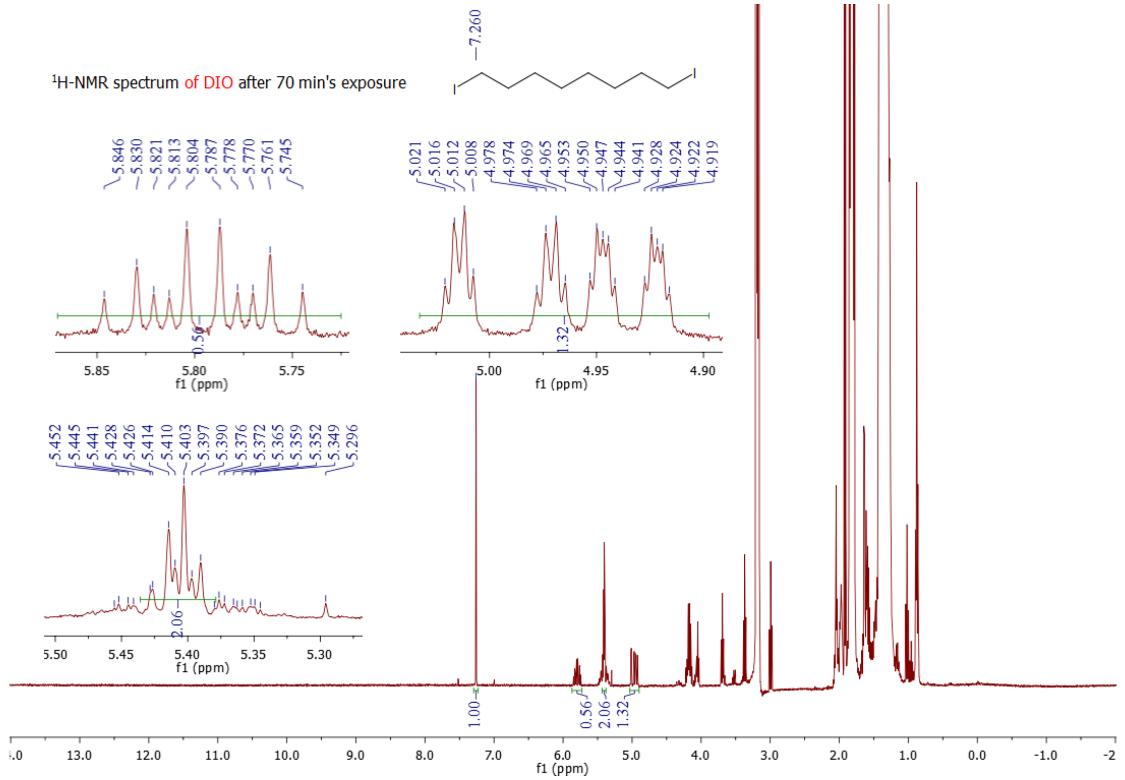

1H-NMR spectrum of DIO after 70 min's exposure
f1 (ppm)

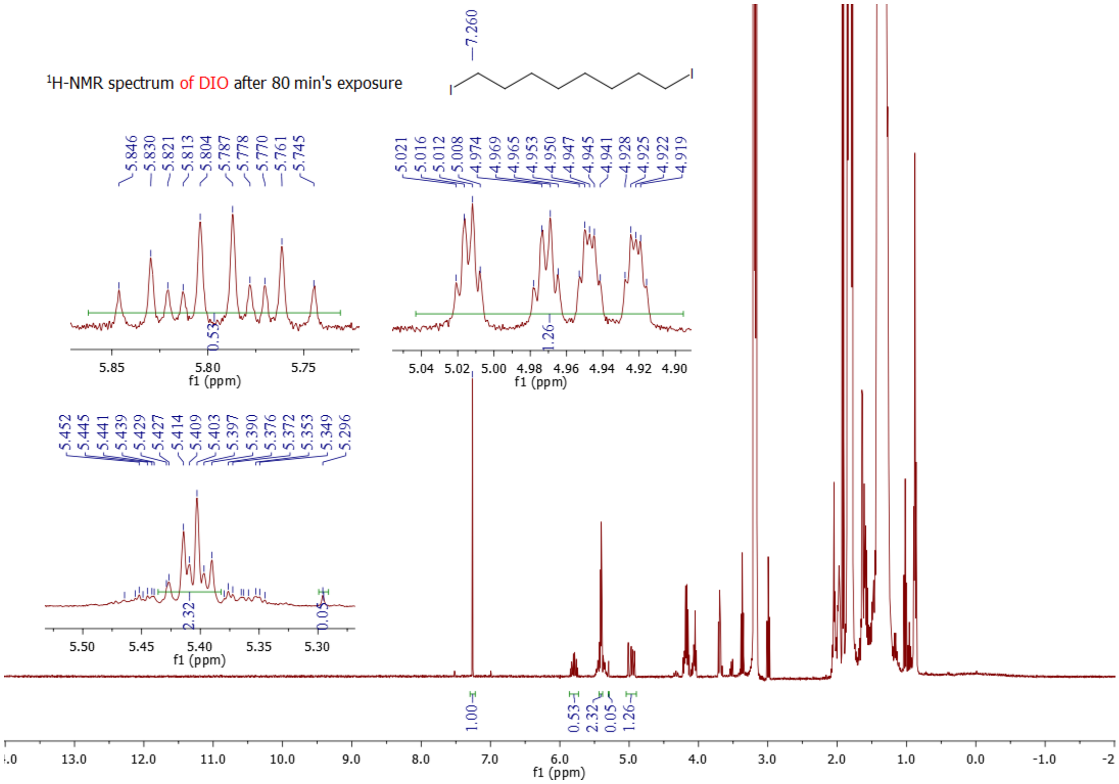

1H-NMR spectrum of DIO after 80 min's exposure
f1 (ppm)

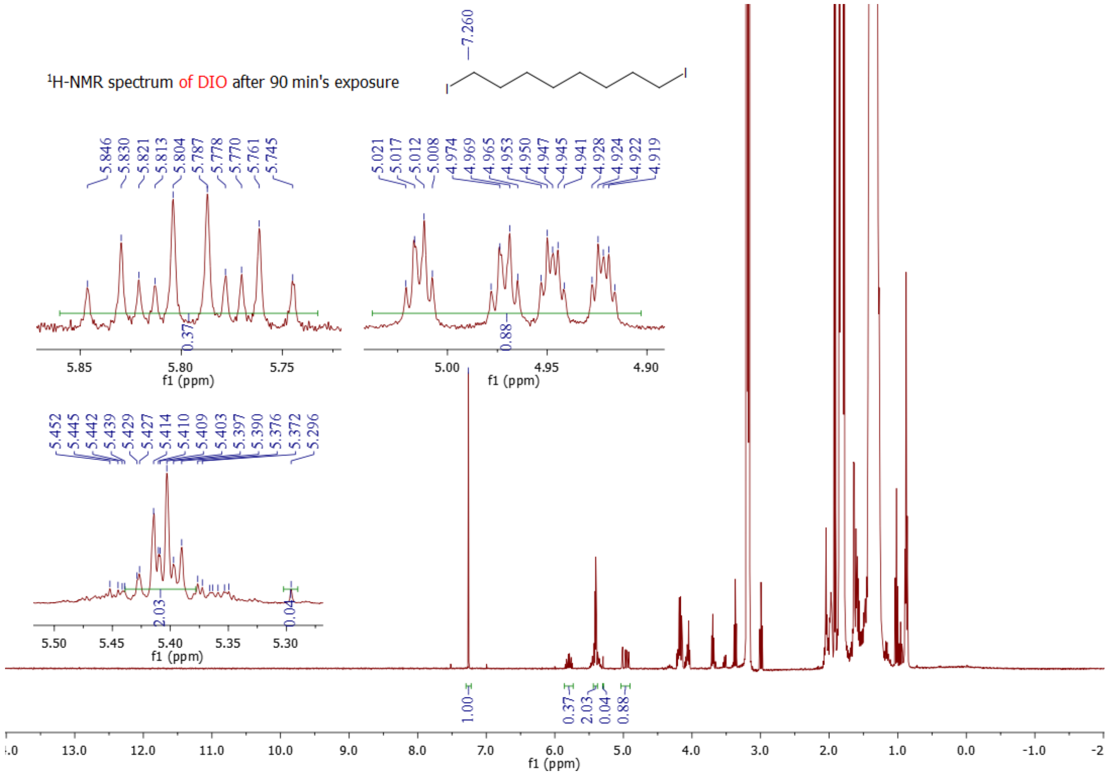

1H-NMR spectrum of DIO after 90 min's exposure
f1 (ppm)

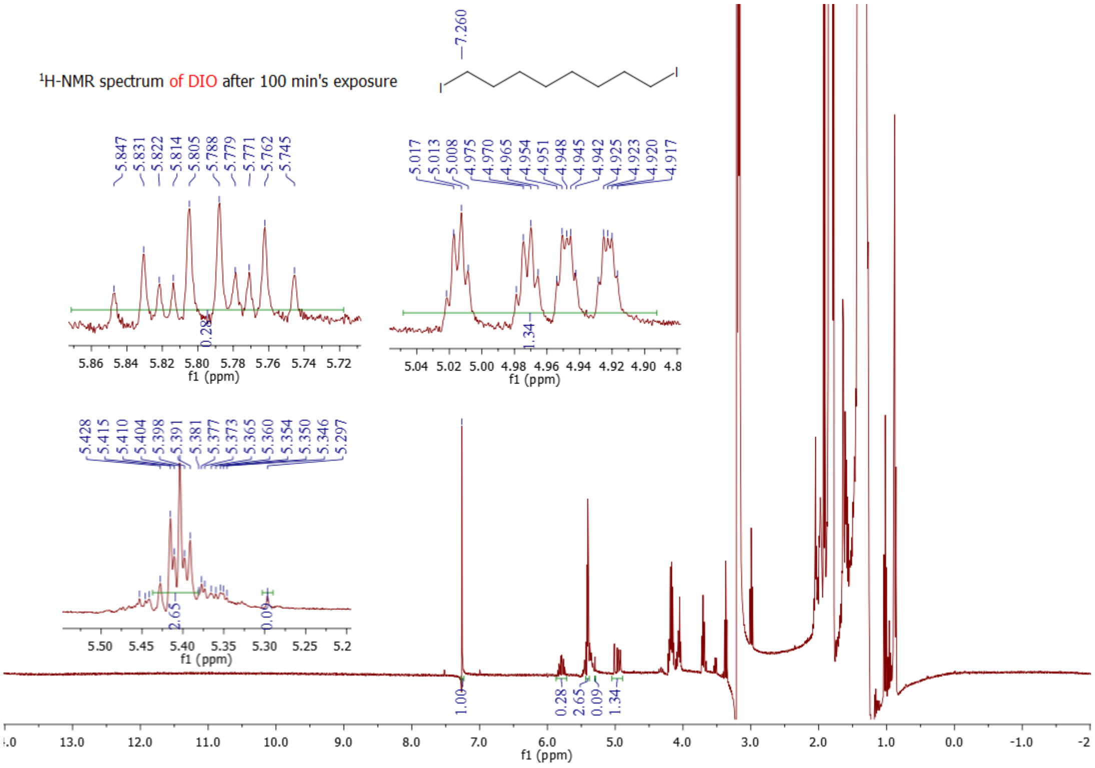
^{1}H-NMR spectrum of DIO after 100 min's exposure
f1 (ppm)

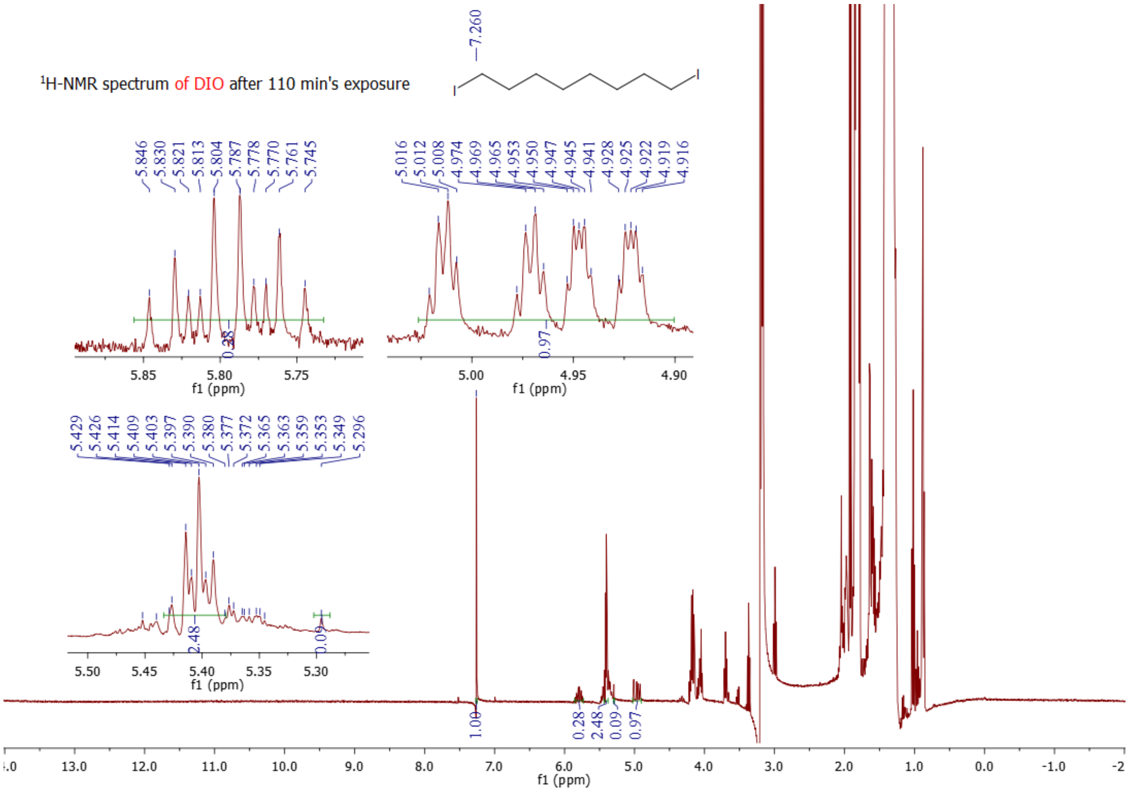
^{1}H-NMR spectrum of DIO after 110 min's exposure
f1 (ppm)

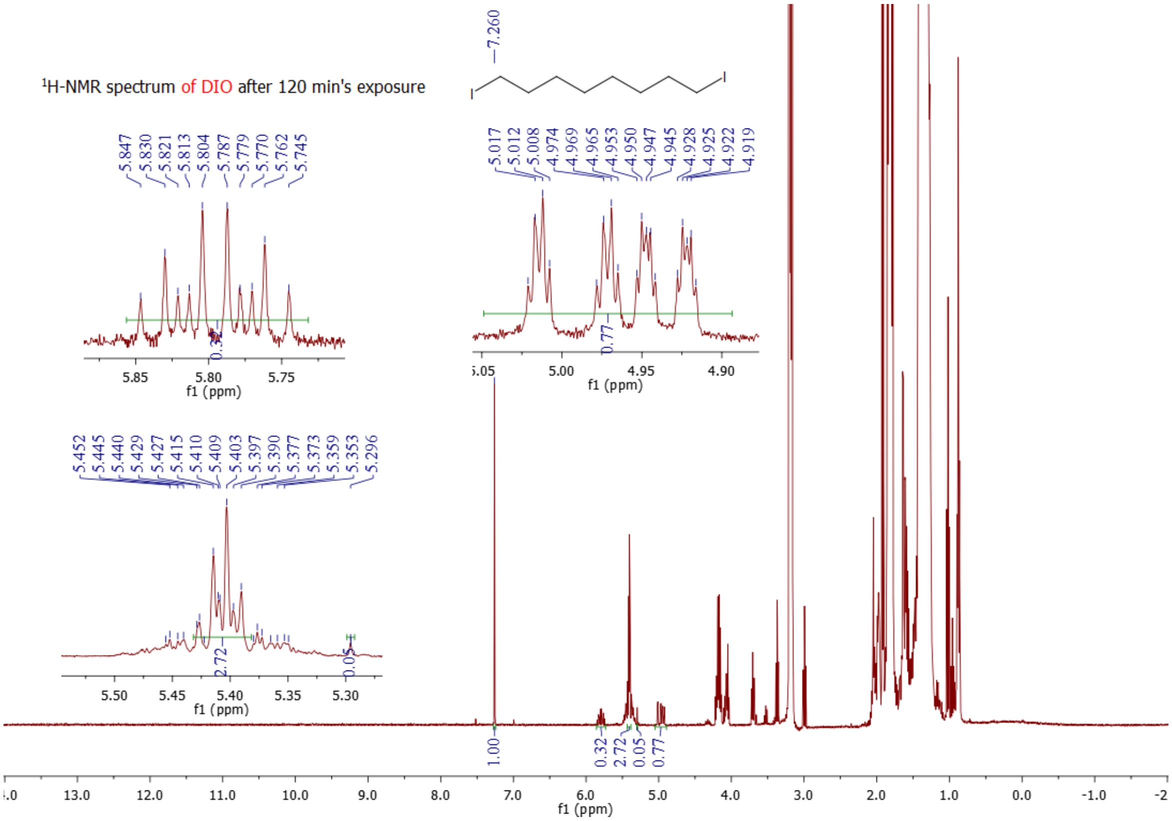

Figure S15. ^{1}HNMR spectra of the DIO-only solution

Original 1H-NMR spectrum with integration of ODT

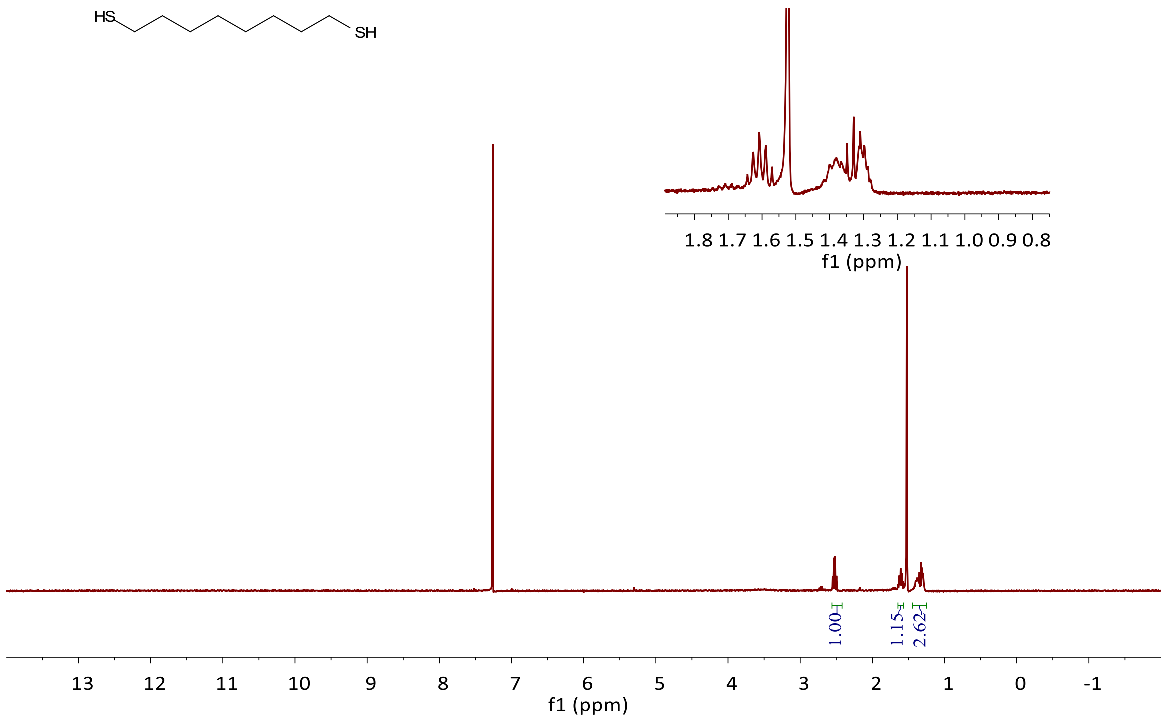

1H-NMR spectrum with integration of ODT after 10 min's exposure

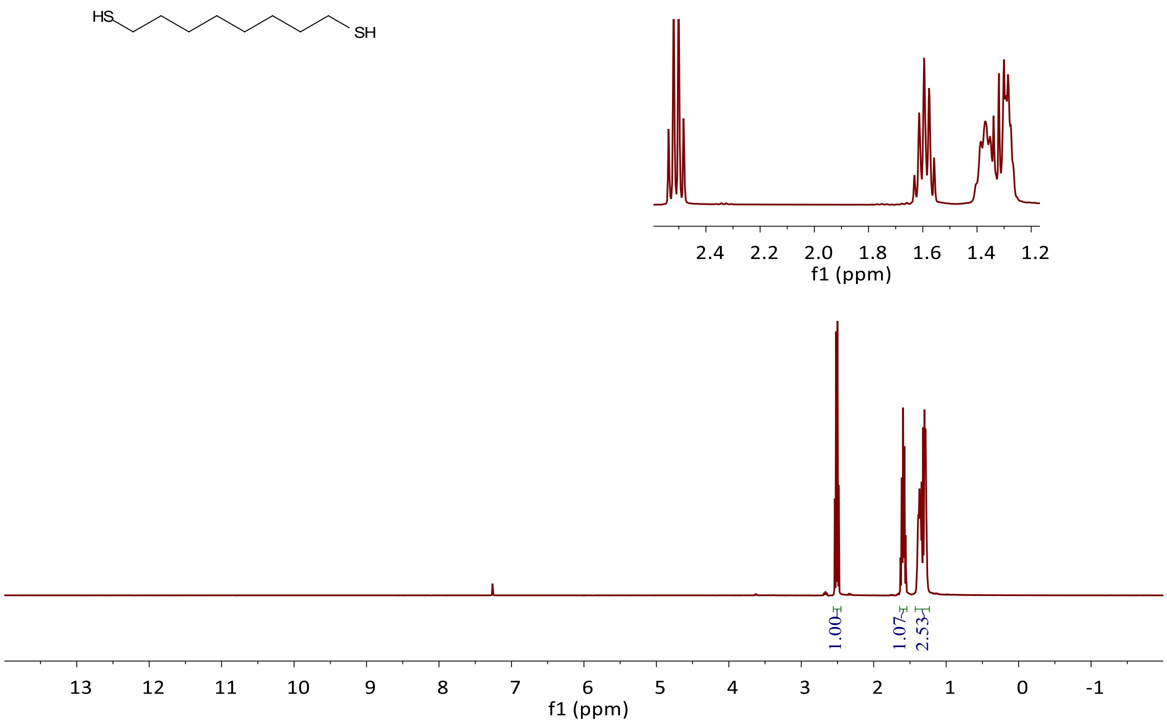

1H-NMR spectrum with integration of ODT after 20 min's exposure

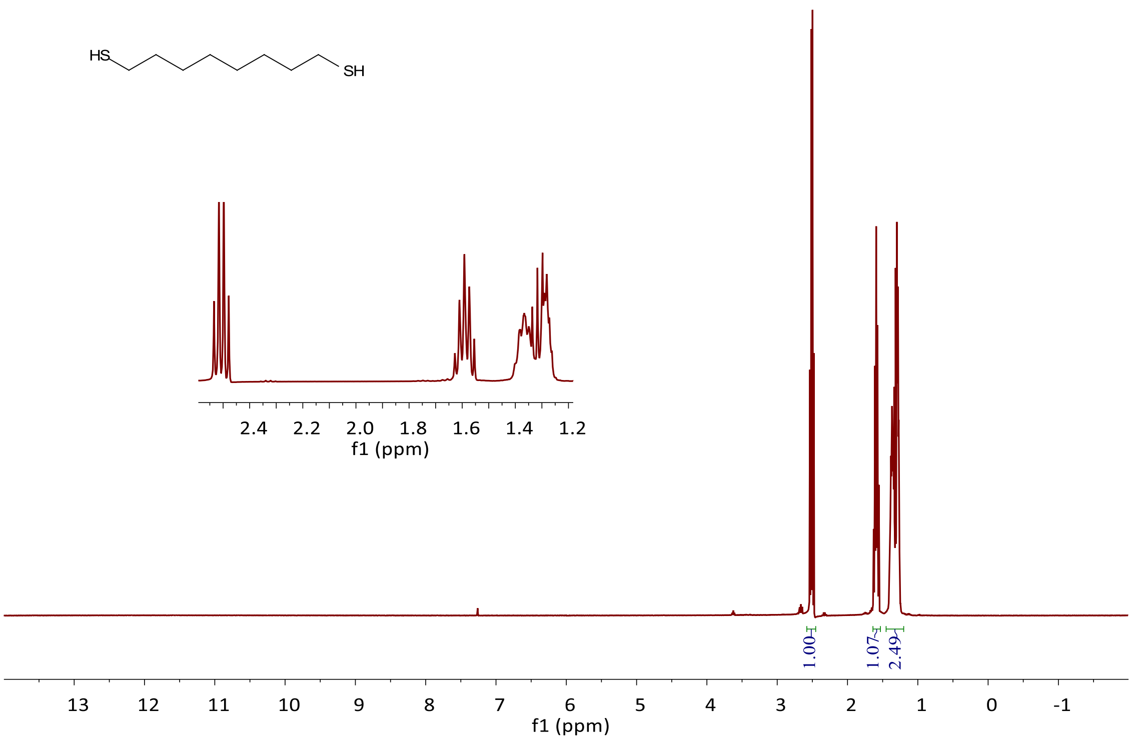

1H-NMR spectrum with integration of ODT after 30 min's exposure

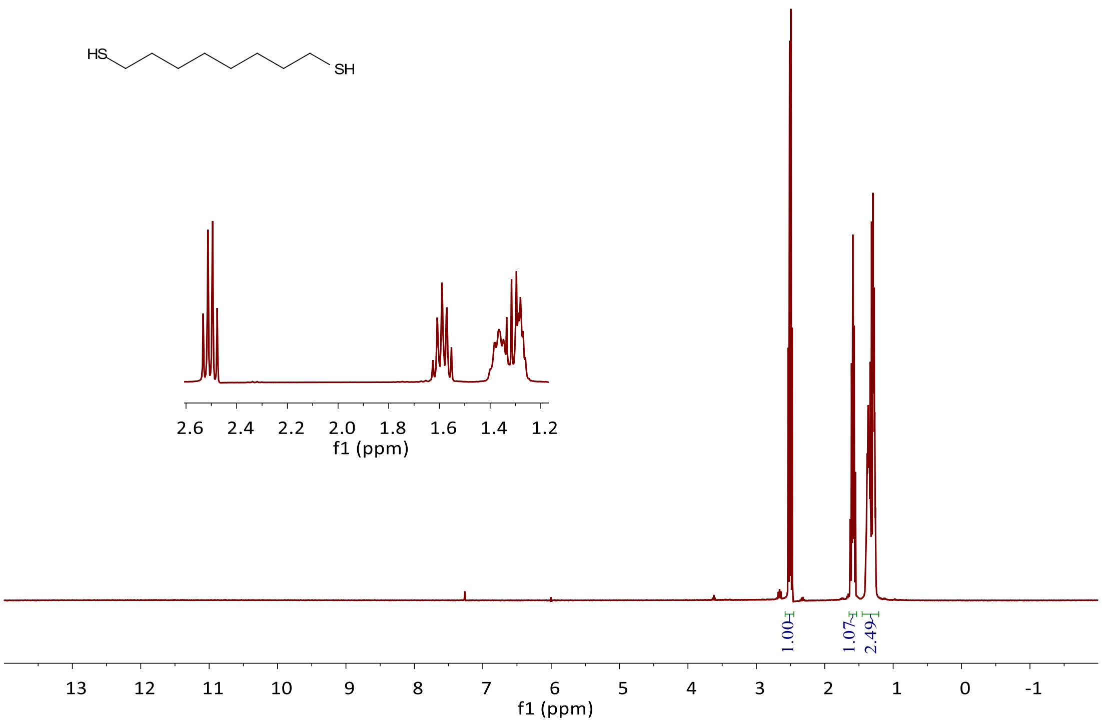

1H-NMR spectrum with integration of ODT after 40 min's exposure

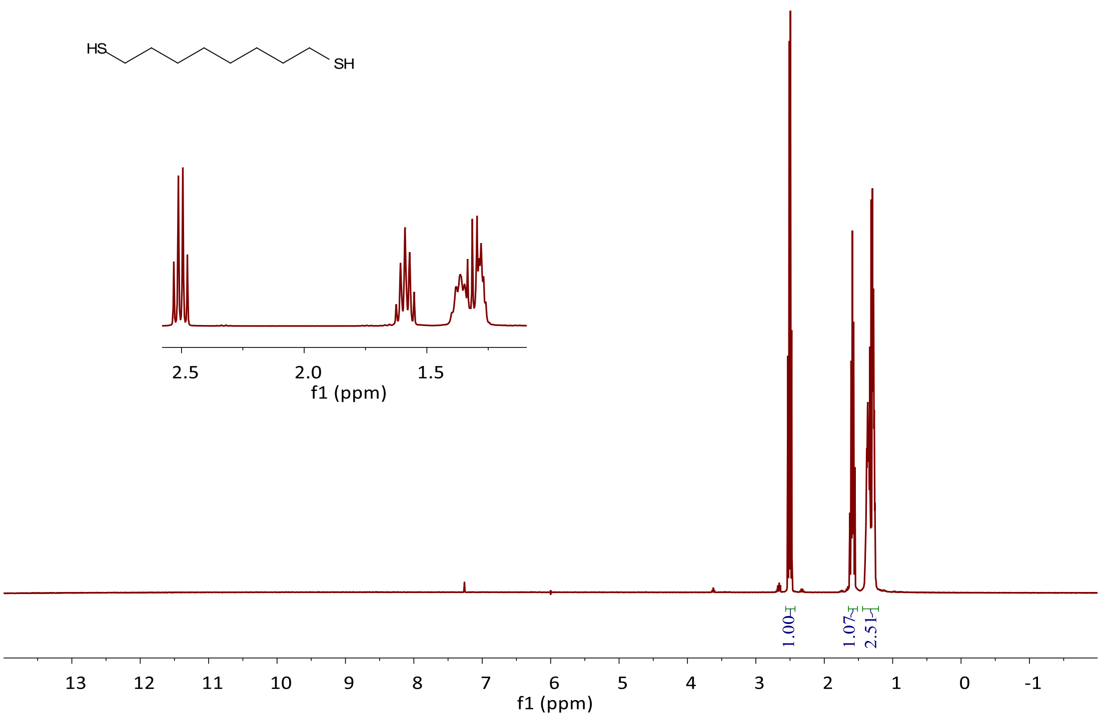

1H-NMR spectrum with integration of ODT after 50 min's exposure

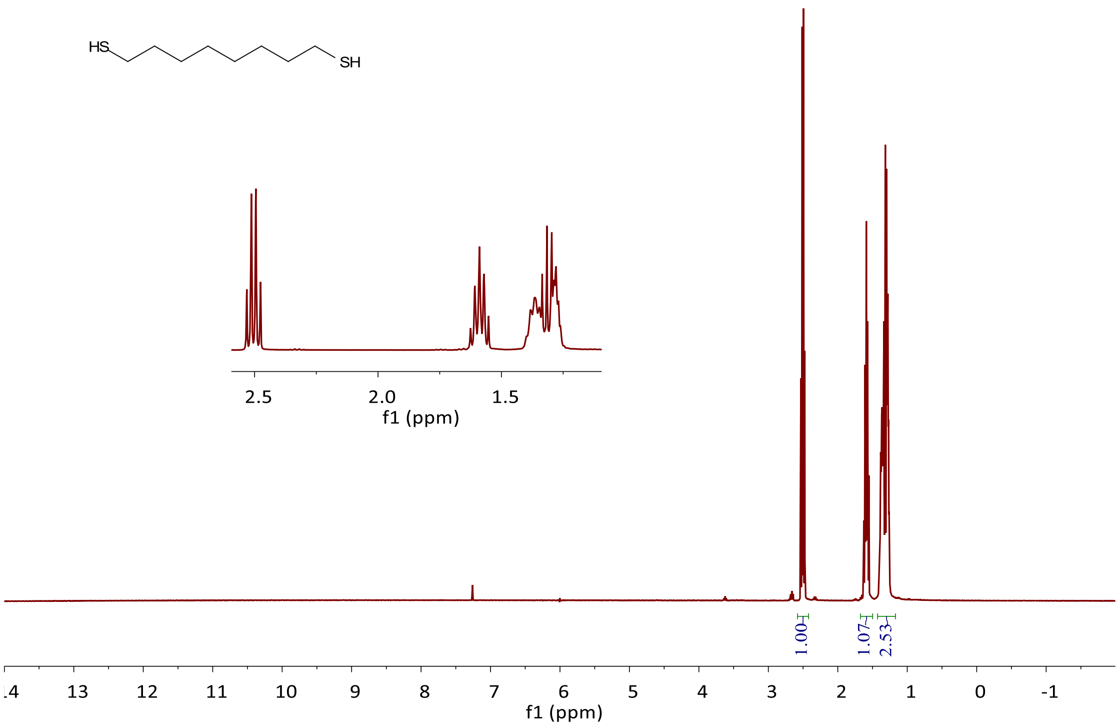

1H-NMR spectrum with integration of ODT after 60 min's exposure

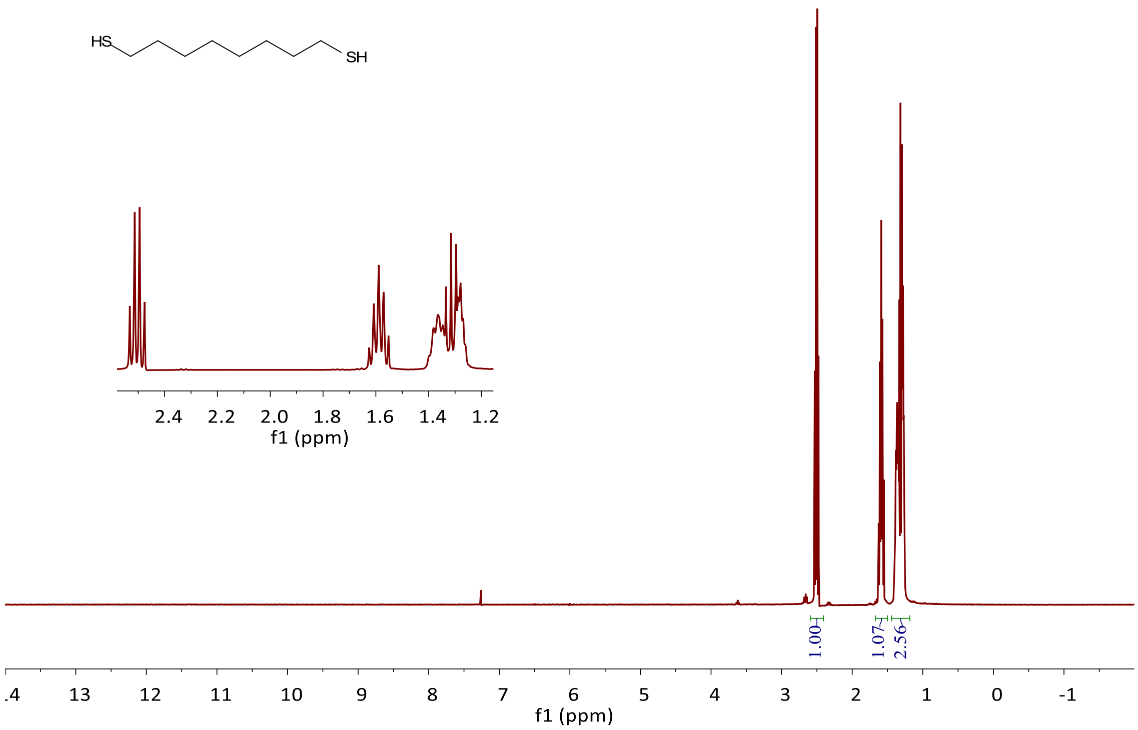

1H-NMR spectrum with integration of ODT after 70 min's exposure

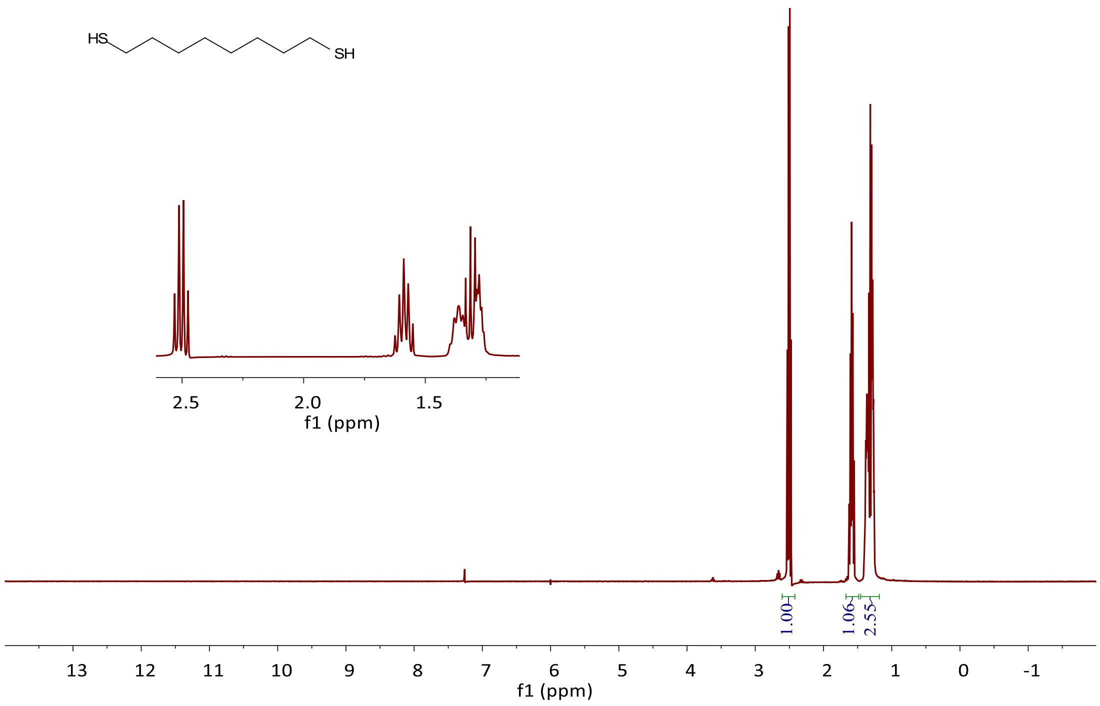

1H-NMR spectrum with integration of ODT after 80 min's exposure

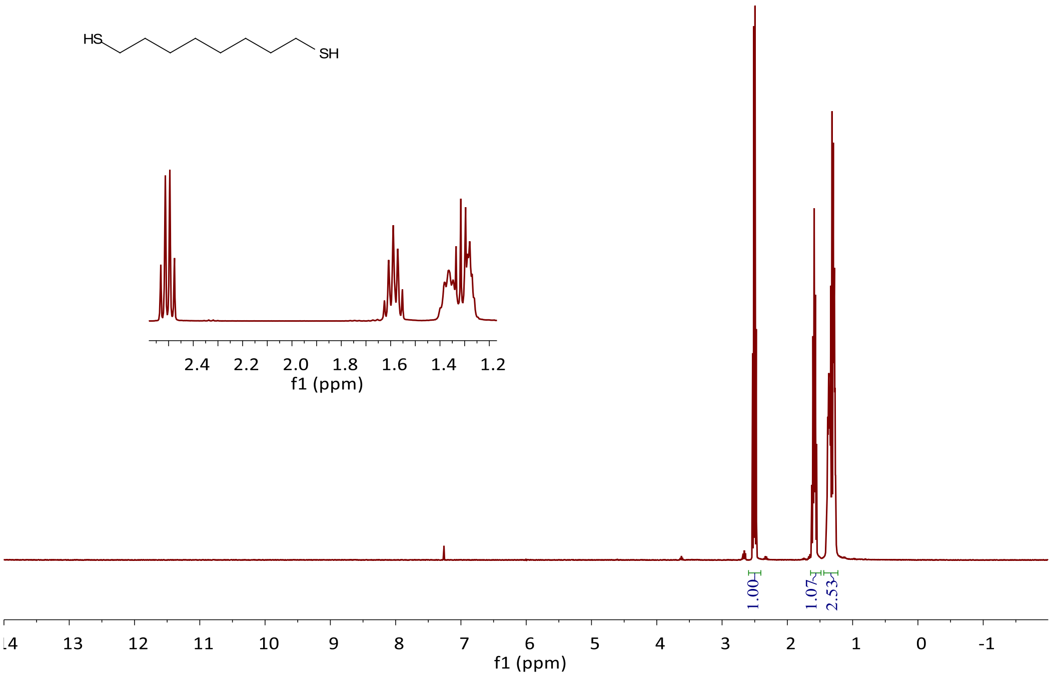

1H-NMR spectrum with integration of ODT after 90 min's exposure

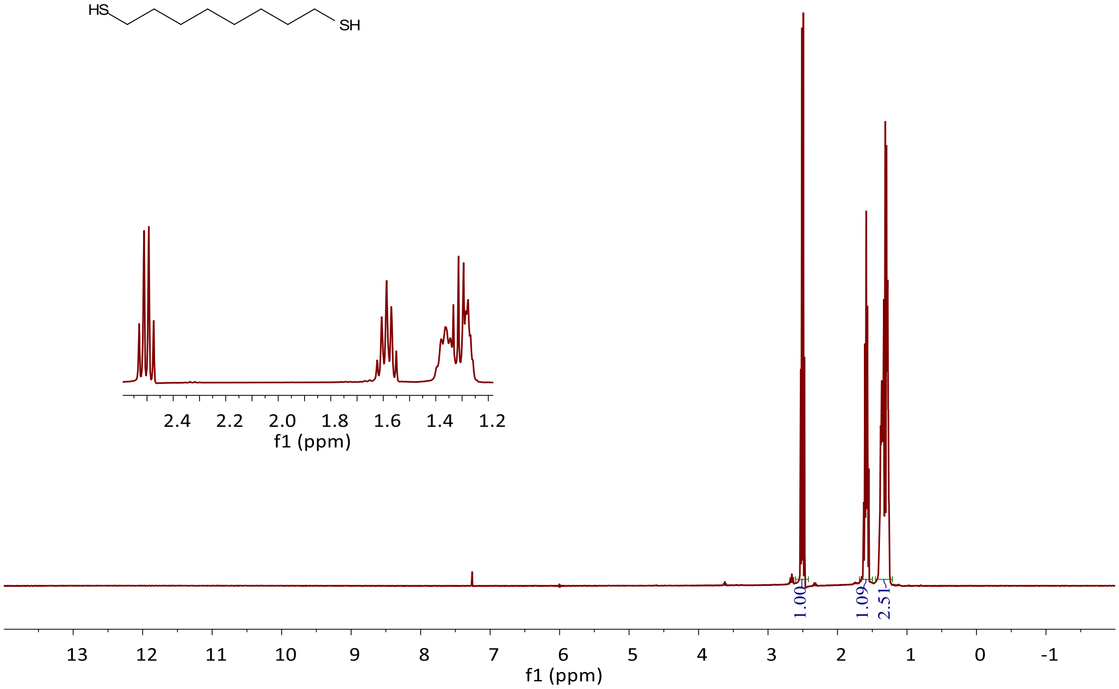

1H-NMR spectrum with integration of ODT after 100 min's exposure

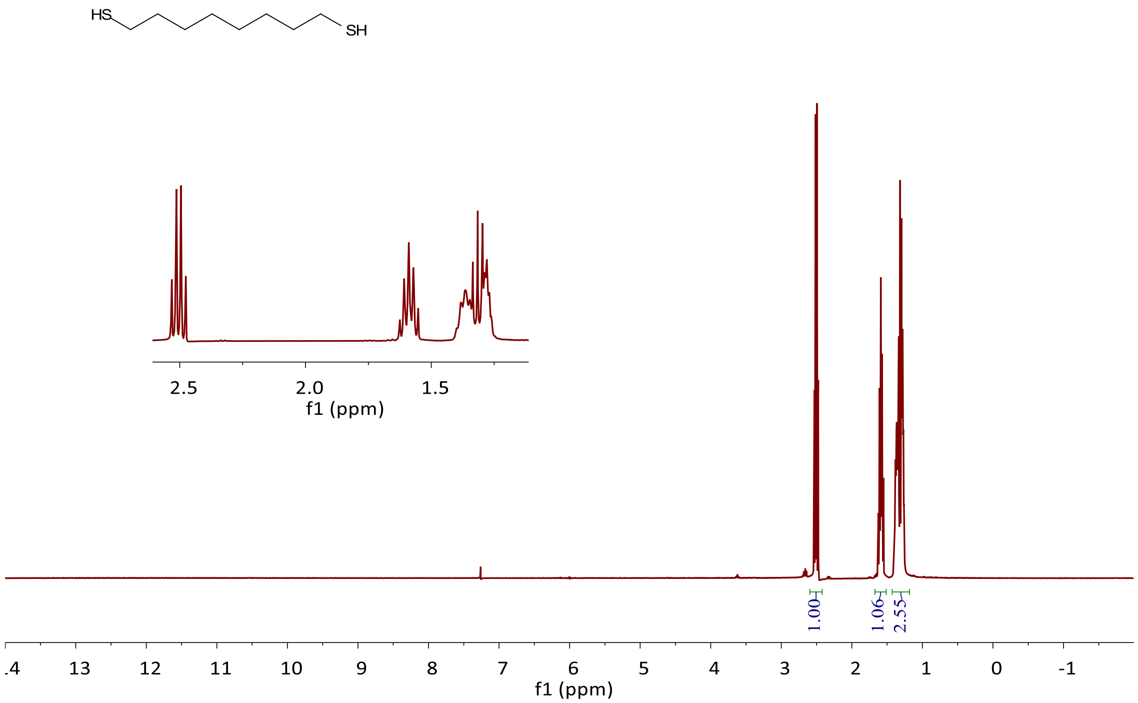

1H-NMR spectrum with integration of ODT after 110 min's exposure

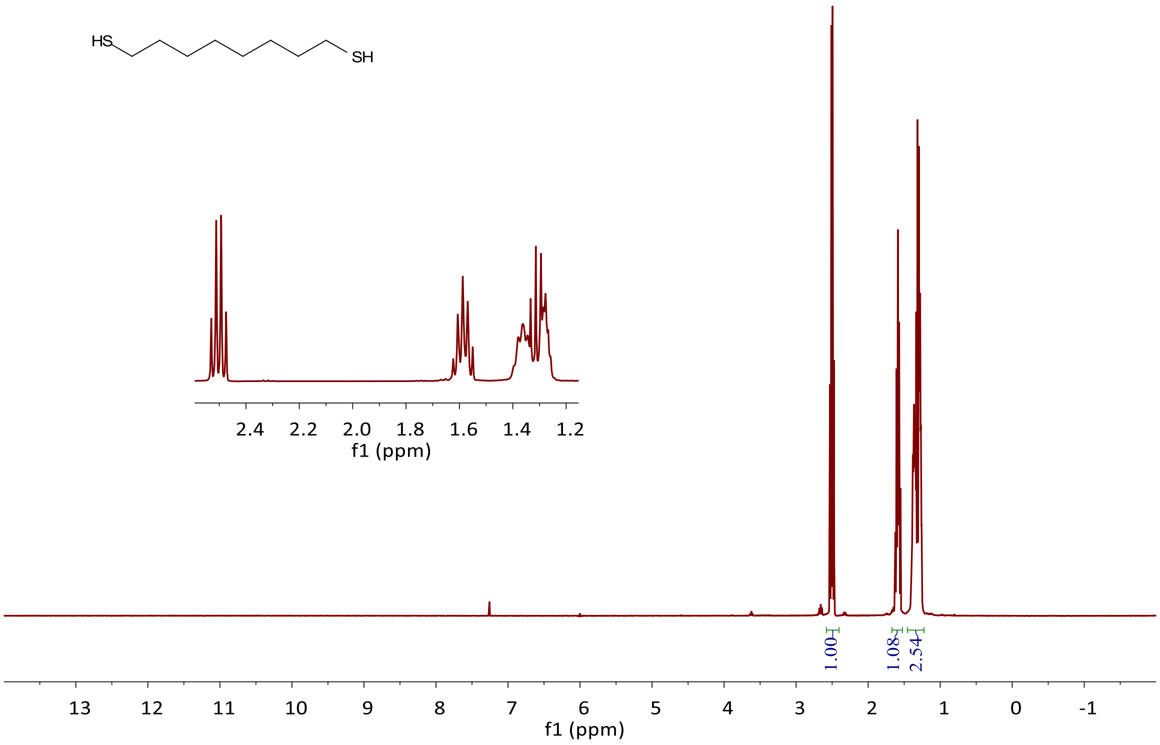

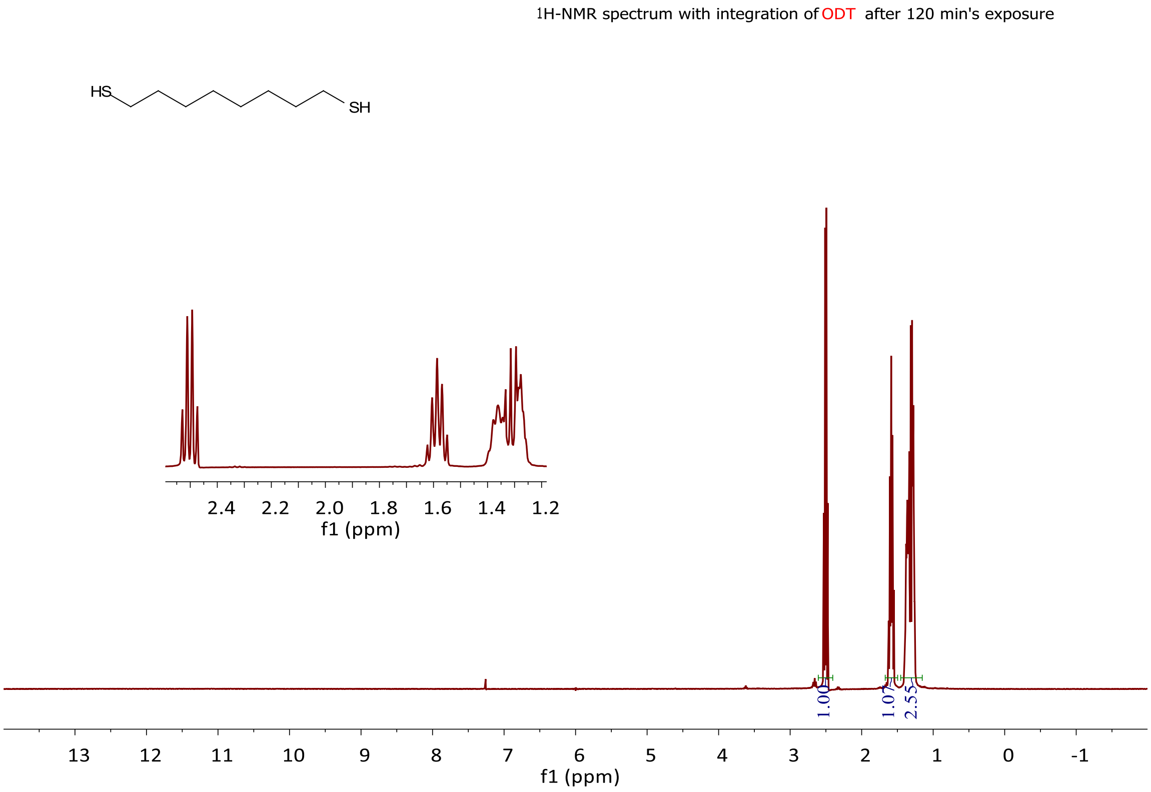

Figure S16. ^{1}HNMR spectra of the ODT-only solution

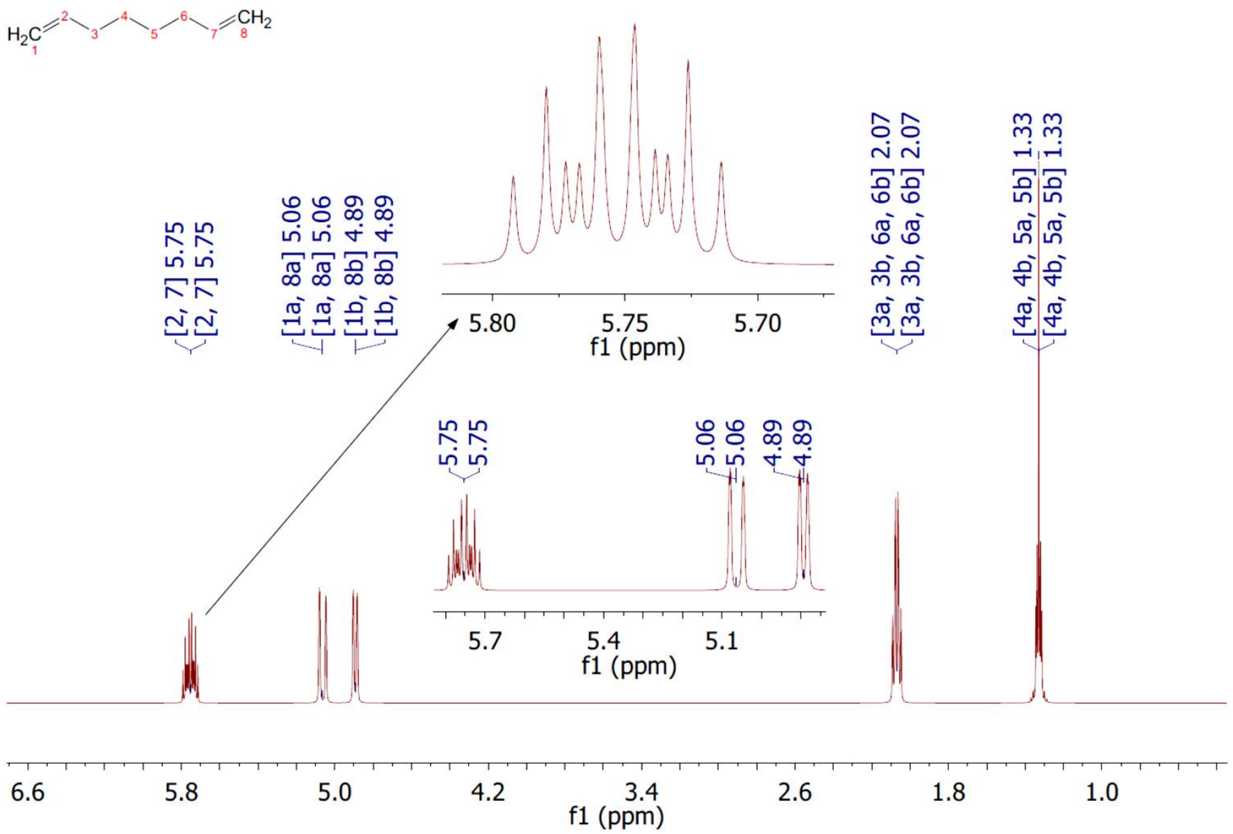

Figure S17. MestReNova prediction oft he ^{1}HNMR spectra of alkene radical left in solution after UV-radiation of DIO very conformed to experimental results in Figure 8 and 9E.

References

1. Bartesaghi, D., Ye, G., Chiechi, R. C. & Koster, L. J. A. Compatibility of PTB7 and [70]PCBM as a Key Factor for the Stability of PTB7:[70]PCBM Solar Cells. *Adv. Energy Mater.* **6,** 1–9 (2016).
